# A Simulation Approach to Assessing Sampling Strategies for Insect Pests: An Example with the Balsam Gall Midge

**DOI:** 10.1371/journal.pone.0082618

**Published:** 2013-12-23

**Authors:** R. Drew Carleton, Stephen B. Heard, Peter J. Silk

**Affiliations:** 1 Canadian Forest Service–Atlantic Forestry Centre, Natural Resources Canada, Fredericton, New Brunswick, Canada; 2 Forest Protection Limited, Lincoln, New Brunswick, Canada; 3 Department of Biology, University of New Brunswick, Fredericton, New Brunswick, Canada; University Toulouse 1 Capitole, France

## Abstract

Estimation of pest density is a basic requirement for integrated pest management in agriculture and forestry, and efficiency in density estimation is a common goal. Sequential sampling techniques promise efficient sampling, but their application can involve cumbersome mathematics and/or intensive warm-up sampling when pests have complex within- or between-site distributions. We provide tools for assessing the efficiency of sequential sampling and of alternative, simpler sampling plans, using computer simulation with “pre-sampling” data. We illustrate our approach using data for balsam gall midge (*Paradiplosis tumifex*) attack in Christmas tree farms. *Paradiplosis tumifex* proved recalcitrant to sequential sampling techniques. Midge distributions could not be fit by a common negative binomial distribution across sites. Local parameterization, using warm-up samples to estimate the clumping parameter *k* for each site, performed poorly: *k* estimates were unreliable even for samples of *n*∼100 trees. These methods were further confounded by significant within-site spatial autocorrelation. Much simpler sampling schemes, involving random or belt-transect sampling to preset sample sizes, were effective and efficient for *P. tumifex*. Sampling via belt transects (through the longest dimension of a stand) was the most efficient, with sample means converging on true mean density for sample sizes of *n*∼25–40 trees. Pre-sampling and simulation techniques provide a simple method for assessing sampling strategies for estimating insect infestation. We suspect that many pests will resemble *P. tumifex* in challenging the assumptions of sequential sampling methods. Our software will allow practitioners to optimize sampling strategies before they are brought to real-world applications, while potentially avoiding the need for the cumbersome calculations required for sequential sampling methods.

## Introduction

Insects and other pests are responsible for enormous financial and production losses in agriculture and forestry. However, pest control can be expensive and often engenders concern over environmental impacts. A central goal of modern integrated pest management is to deploy pest-control interventions as efficiently as possible, in order to reduce crop damage at minimum cost and with minimum collateral damage to the environment.

Perhaps the most basic requirement for any pest management program is the availability of a sampling method for assessing the level of infestation (either estimating mean pest density, or judging whether density exceeds a threshold beyond which intervention is deemed necessary). For simplicity, in this paper, we use vocabulary associated with insect pests, although our discussion is equally applicable to other types of pest. Estimating insect densities in the field is far from a simple task, and it involves decisions about when to sample during host or insect phenology (e.g., [1]), what to sample (quadrat, whole plant, appropriate organ, or representative module; e.g., [2]), and which and how many plants, or other sampling units, to sample from the large number available at a site. This last decision in particular has spawned an enormous literature [3], with thousands of idiosyncratic recommendations for different systems but with a simple underlying truth: in general, more accurate estimation is achieved by including more samples and selecting them in more sophisticated ways; but doing so requires more time, money and labour. Achieving the most accurate estimates from the smallest investment of effort can involve ingenuity in field technique (e.g., [4]), but great returns can also come from the development of statistical methods for handling sampling data and for evaluating the efficiency of alternative sampling designs (e.g., [5]–[7]).

One important technique for efficient estimation is sequential sampling, which is widely applied in agriculture and forestry [8]. In sequential sampling, samples are added to a data set one by one, with a check after each addition to determine whether the data set yet allows sufficiently strong inference about infestation. This approach promises large savings in sampling effort because it can identify (in real time) the point when further sampling would return too little additional information to merit its cost. These savings in effort carry, however, a potential cost: decisions about when sampling can stop are based on calculations that assume considerable information about the distribution of insects across sampling units.

### Parameterization requirements for sequential sampling

The key to a sequential sampling scheme (whether designed for estimating density or evaluating density against a threshold) is a “stopping rule” that formalizes the decision to continue or stop sampling after each new sample is taken. For estimating density, the stopping rule takes the form “stop sampling if a confidence interval around the estimate is narrower than X”. For decisions about density thresholds, the stopping rule takes a slightly more complex form: “stop sampling if the cumulative insect count for *n* samples is above *f_1_(n)* or below *f_2_(n)*”. The functions *f_1_(n)* and *f_2_(n)* are specified such that a count above *f_1_* indicates confidence that the true density exceeds the density threshold, a count below *f_2_* indicates confidence that the true density is below the density threshold, and any other count indicates inability to decide.

Specification of these stopping rules depends on the ability to fit insect densities to known distributions with well-estimated parameters [3], [8]. Sequential sampling methods can take one of two approaches (single or local parameterization) depending on the level of local detail to be incorporated. The most common approach, single-parameterization sequential sampling, proceeds by assuming that the distribution of insect densities across sampled plants has an unknown and spatially variable mean (*μ*) but otherwise can be fit everywhere by a single set of parameters. For example, an insect's densities might be well represented everywhere by a normal distribution with a common *σ^2^* (variance), or by a negative binomial distribution with a common *k* (clumping parameter). Once these parameters are known, they can be used to generate a universal stopping rule to be applied to the estimation of *μ* in all studied populations (“Wald's procedure”; [3], [9], [10]). Alternatively, with data from enough sites, among-site variation in insect distributions can itself be parameterized, for instance by fitting a power law [11] to describe the relationship between local mean and variance. This parameterization can then be used to calculate a stopping rule incorporating local variation, albeit at the cost of some added complexity [12], [13].

Single-parameterization methods offer practical assessment tools that demand only moderate mathematical ability of practitioners in the field. Unfortunately, though, the assumption that a single parameterization can be applied to every population of a given insect is frequently violated. Instead, it is common for not just mean density but also the form of an insect's density distribution to shift in space (e.g., [14], [15]), in time (e.g., [16]), or in response to changes in the resource landscape (e.g., [17]). In principle, this problem can be overcome by local-parameterization sequential sampling: the application of Wald's procedure, but with a preliminary step in which distributional parameters such as *σ^2^* or *k* are estimated separately for each local site. The most straightforward method involves taking, at each site, a warm-up sample of *n_0_* plants to estimate local *k* (or other appropriate parameter(s)); this parameter estimate is used in turn to calculate a stopping rule for density estimation specific to that site. Data for the warm-up sample can be re-used as the first *n_0_* plants in sequential sampling, or with a more sophisticated approach, the parameterization step can be integrated with sequential sampling so that parameter estimates are refined as sampling proceeds [13], [18]. Local-parameterization procedures can accommodate variation in insect distribution across sites, but at the cost of using complex stopping rules that cannot be specified in advance of sampling a site.

### Assessing the likely performance of sequential sampling and alternatives

The high efficiency promised by sequential sampling may not always be realized. Parameterization may fail outright (for instance, if distributional parameters vary even within sites), within-site spatial autocorrelation may make even local parameterization misleading [19], or the warm-up sampling effort needed to parameterize distributions may be prohibitive. The latter problem is especially likely to arise for insects with aggregated distributions, because stopping rules depend on aggregation parameters (for instance, the negative-binomial *k*) that can be very difficult to estimate from field data [20]–[22]. Ironically, in the pest-control context, *k* is generally only a nuisance parameter: its value is needed for sequential sampling, but it is not intrinsically important to decisions about intervention. These decisions are usually based instead on mean insect density, and means are much more easily estimable. As a result, it is possible for the warm-up sampling effort necessary in advance of sequential sampling to exceed the effort necessary for decision making itself.

In this paper, we develop new tools for assessing the feasibility of sequential sampling for a particular pest system, and furthermore, for assessing the performance of alternative sampling strategies for insect pests. Use of these tools will allow the deployment of sequential sampling when it can deliver savings in overall sampling effort, while recognizing cases where alternatives outperform sequential sampling: for instance, when adequate estimates of mean density can be made with sample sizes too small for good estimates of nuisance parameters like *k*. Our methods take advantage of computer simulation, given the availability of pilot density data for a set of sites sufficient to be representative of both within-site and among-site variation in insect distribution. We will refer to these pilot data as a pre-sample (to distinguish the pre-sample, taken once, from warm-up samples taken for every site where density is to be estimated, as in local-parameterization sequential sampling). Of course, the requirement for a pre-sample means that we cannot entirely escape the need for sampling in advance of density estimation. However, there are at least three potential advantages to performing a single bout of pre-sampling rather than taking warm-up samples every time estimation is desired. First, investment in pre-sampling effort may reduce total effort in the long term, if we learn that we can avoid ongoing warm-up sampling for a given system. Second, pre-sampling, and the analysis of data from the pre-sample, can be conducted by specialized personnel, allowing practitioners such as farmers or woodlot owners to follow simpler sampling procedures with a lower computational burden. Third, pre-sampling data can be used to consider a wide range of alternative sampling schemes: in addition to determining efficient sample sizes, we can assess the efficiency of different estimation procedures and different ways to select sampling units, such as random vs. transect sampling.

We illustrate our approach with data for the balsam gall midge, *Paradiplosis tumifex* Gagné (Diptera: Cecidomyiidae), an insect pest of Christmas tree farms, using a data set from seven farms in New Brunswick, Canada. We ask whether *P. tumifex* distributions are homogeneous among sites (permitting single-parameterization sequential sampling) or at least can be easily parameterized at each site (permitting local-parameterization sequential sampling). We show that neither condition is met and we therefore use a simulation approach to assess alternative sampling strategies. We demonstrate efficient methods for density estimation and threshold decision making for New Brunswick *P. tumifex* and we provide software with which our approach to assessing sampling strategies can be applied to other systems.

## Methods

### Study system: Balsam gall midge in Christmas tree stands

In eastern Canada, the sale of Christmas tree and wreath products from *Abies balsamea* (L.) Mill. (balsam fir) is a multimillion dollar industry, with trees shipped to markets throughout the western hemisphere [23]. Among major pests of Christmas tree crops is *Paradiplosis tumifex* (balsam gall midge), a univoltine, needle-galling cecidomyiid that attacks balsam fir across the tree's natural range. Most needles galled by *P. tumifex* turn yellow and fall from the tree in the year of attack, with defoliation most severe high in the crown [2], [24]. The natural history of *P. tumifex* is further described by [25].

At low densities, *P. tumifex* is of little consequence for Christmas tree farmers. However, populations can build rapidly (1–2 years) to levels causing 80–90% defoliation of the upper crown (D. Carleton, pers. obs.). Such substantial defoliation can significantly reduce the tree's photosynthetic capability and growth rate and alter patterns of shoot development [26], [27]. In marketable-size trees, defoliation reduces aesthetic appeal and thus suitability and/or value for sale. Although farmers vary in their tolerance for *P. tumifex* damage, most would consider mid-crown infestation around 1% (of needles galled) to be low, with 5% being moderate, and 10% a high level of infestation clearly meriting intervention (M. Wright, Nova Scotia Christmas Tree Farmers' Association, pers. comm.). Given the potential for financial loss from *P. tumifex* attack, farmers would benefit not only from an efficient way to assess infestation before they decide whether to deploy control methods, but also from an efficient way to assess the level of control achieved after intervention. The need for such techniques will only become more acute, as regulatory changes aimed at reducing pesticide use mean that new control strategies will need to be developed and assessed: only one pesticide is currently registered for *P. tumifex* in Canadian Christmas tree farms, and it is listed for long-term phase-out under the Pesticide Management Regulatory Act.

For several reasons, *P. tumifex* is a good case study with which to illustrate our methods for assessing sampling strategies. First, despite the pest's importance, no practical monitoring program has been available for *P. tumifex*. Giese & Benjamin's [24] recommended sampling schemes were labour-intensive and impractical for application by growers. Only recently has a functional sampling unit been determined at the tree level [2], and no formal analysis has been available to guide site-level density estimation or decision making with respect to pesticide-application thresholds. Second, the development of comprehensive pest management strategies for *P. tumifex* (and for other Christmas tree pests) is further hampered by a high diversity of agricultural practices in the industry. Farms can range in size from <1 ha to >100 ha and are derived from reclaimed agricultural fields, forest clearcuts, and even disused military compounds. Farms can be bordered by pastures, row crops, water, or forests. Seedlings for tree stock can come from natural regeneration, sowing of purchased seed stock, out-planting of seedlings or combinations of the three. Pest management practices, including willingness to use insecticidal sprays and methods used to assess pest density, are highly variable among farmers. This diversity means that any monitoring strategy must be robust enough to deal with substantial variation in attributes of sites, crop, and farming techniques. Finally, we suspected (based on previous observations) that *P. tumifex*, like many other insects, would possess complex distributions that could make conventional sequential sampling inefficient or ineffective.

### Field sites and sampling methods

We surveyed *P. tumifex* infestation in seven Christmas tree farms ( = sites A, B, C, D, E, F, and G) in central New Brunswick from 11 July–1 August 2012. Permission for land use for the purpose of this research was approved by the Christmas tree growers (see Acknowledgements) on their private lands. Neither the land used nor any insect species sampled was designated as protected, and as such no permits were required. Ethics permission is not needed for insect-related experimentation. We chose our sites because they had known midge infestations and were close enough together for convenient sampling, yet included owners who use a broad range of agricultural practices. At each site, we sampled either 100 (sites A and F) or 200 (remaining sites) trees depending on stand size, selecting trees of marketable size (i.e., saleable within the next two years) in a grid pattern at ∼10 m spacing. Maps of sampled trees for all sites are provided in Figure S1. Tree positions were recorded using a Garmin 600cs and Garmin BaseCamp software (version 3.2.2; Garmin International, Inc., Olathe, KS, USA) to 3 m accuracy. We converted GPS coordinates from degrees latitude and longitude to north and east distances in metres from a point near the centre of our study area (46°N, 66°W). We used sampling methods prescribed by [2] to assess *P. tumifex* infestation. Briefly, for each sampled tree, we collected terminal shoot clusters from one south-facing, dominant mid-crown branch. We recorded shoot length and number of galls for each shoot and estimated the percentage of galled needles per shoot cluster. This estimate was based on the total number of galls counted, divided by an estimate of total number of needles from regressions of actual needle count on shoot length for 100 mid-crown shoots from each site. Our sampling produced a data set with *P. tumifex* density estimates for 100–200 mapped trees at each of seven sites, with two alternative density measures (total gall count and percentage of needles galled). We used this data set, for which we can easily calculate the actual mean infestation, to assess the performance of alternative sampling schemes that considered subsets of the full data.

### Analyzing *P. tumifex* distributions

We began data analyses by assessing the fit of our *P. tumifex* data to standard statistical distributions. Of our two measures of infestation for each sampled tree, the number of galls (a count) is simpler, but the percentage of galled needles (a continuous variate) is of more direct importance to both host plants and farmers. Separately for each site, and using our full sets of trees, we tested the fit of each measure to normal distributions using the Shapiro-Wilk test in R version 2.12.0 [28]. We fitted the gall count data to negative binomial distributions (again, separately for each site) using the ‘fitdistr’ function of R package ‘MASS’ and then tested for goodness-of-fit using the ‘goodfit’ function of R package ‘vcd’. We also rounded the percent galling data to the nearest 1%, making a pseudo-count variable that we tested similarly for fit to the negative binomial. Because fitting rounded percent galling to a negative binomial gave by far the best fits, we used this measure and distribution for all further analyses.

We then asked how well we could estimate *k*, the clumping parameter of the negative binomial distribution, based on smaller samples of infestation data. We used a script in R to draw, randomly and with replacement, infestation data for *n* = 20, 50, or 100 trees from the larger data set for each site, and to estimate *k* for each draw (again using the ‘fitdistr’ function). We made 100 such draws for each sample size at each site. We then plotted the *k* estimates and calculated intervals containing the central 50% and 90% of the estimates; when these confidence intervals are narrow, estimation is performing well.

We tested for spatial autocorrelation in infestation rates within each site using function ‘mantel.rtest’ of R package ‘ade4’. We visualized spatial pattern via semivariograms using function ‘variog’ of package ‘geoR’ in R. All of our R scripts are provided (Software S1).

### Simulated sampling

The distributional complexity revealed by the foregoing analyses motivated us to explore alternative approaches to estimating *P. tumifex* infestation. We simulated sampling using several different rules for ordered drawing of trees from the larger data set for each site: (1) random sampling; (2) ordered sampling by collection number; and (3) ordered sampling by belt transects. For random sampling, we executed 10,000 randomizations for each site, whereas ordered samplings were deterministic. Simulations were implemented using InfestSample version 1.10, written by SBH in Microsoft Visual Basic.NET for Windows. This software is available as a zipped executable (Software S2), as a source-code text file (Software S3), as a zipped Visual Basic project folder (Software S4), or from github.com (user stephenbheard).

Our random sampling procedure drew trees with replacement from the larger data set for each site (Figure S2A). We sampled with replacement because our original field sampling included only trees >10 m from their closest sampled neighbours, rather than all trees present, and so our “full” data set is in turn a sample from a larger statistical population of trees. Random sampling is motivated by the usual expectation that it should provide unbiased estimates of population parameters.

Ordered sampling by collection number included trees in the same order as they were encountered in our original field sampling. In each case, this meant a back-and-forth raster starting at one corner of the stand, sampling along an edge, moving 10 m deeper into the stand and returning parallel to the edge, and so on until the entire stand had been sampled (Figure S2B). This scheme is motivated by the expectation that future sampling crews might choose to visit trees across the stand in a convenient order, as did our original field crews. We also considered backward sampling by collection number, which simply reverses the first order.

Ordered sampling by belt transect included trees as encountered along a series of parallel belt transects through the stand (Figure S2C). The simulation software permits user selection of start and end points for one or two transect sets. Two transect sets may be used for sites that include sub-site structure: for instance, our sites A and C each comprised a larger and a smaller stand separated by some distance (Figure S1), and we used two transect sets for each site. For each transect set, sampling includes trees from the overall site sample that fall within a belt transect of width *w* laid out between the specified start and end points. Trees are added to the sample in the order they are encountered along the transect. Following completion of the first transect, a second and third transect are added to the transect set using a “jitter” of length *j*: the second transect is parallel to the first but with its centre *j* metres to the left, and the third is *j* metres to the right. When there are two transect sets, they are interleaved such that set A's first transect is followed by set B's first transect. For our sites, we specified start and end points corresponding to trees marking opposite ends of the stand in its longest dimension. We used transects of width *w* = 10 m, with jitters of *j* = 20 (sites A, F), 30 (sites C, D, E, G), or 40 (site B) m, setting *j* to spread the transects out across the breadth of the stand. We also considered sampling along the transects in reverse order. Belt transect sampling was motivated by the notion that such transects provide relatively easy field sampling while tending to cut across within-site spatial variation in the density being estimated.

For each simulated sampling approach, we added trees one at a time to the sample (*n* = 1, 2…‥*N*, where there are *N* = 100 or 200 trees in the full site sample). For each *n*, we estimated site mean infestation and calculated the absolute deviation of that estimate from the “true” mean (that estimated from the complete site sample). For random sampling, for each *n*, we also calculated 95% confidence intervals around the mean estimate and percent correct decision rates for comparisons of the estimated infestation with threshold infestations of 1, 3, 5, 7, and 10%. A decision is correct for the 5% threshold (say) if the estimated infestation for *n* trees and the true infestation are both above or both below 5%, and it is incorrect otherwise. For ordered sampling approaches, which are deterministic, confidence limits and correct decision rates are not defined.

## Results

### Pre-sampling

Our seven sampled Christmas tree farms experienced *P. tumifex* attack ranging from 1% to 7% of needles galled in the mid-crown (Table 1). Because attack and defoliation are more severe in the upper crown than in the more easily sampled mid-crown, this range of attack rates includes moderately severe infestations that would provoke pesticide intervention from most farmers. These estimates are exact when applied to the N = 100 or 200 trees in our pre-sample, but have uncertainty if viewed as bootstrap estimates of infestation for the entire farm. We calculated precision as half the width of the 95% confidence envelope divided by the estimated infestation (Table 1). Precision ranged from ±12% to ±23% (for the farms with smaller N), which we consider acceptable performance for our pre-samples in estimating whole-farm infestation. However, for simplicity, in what follows we will refer to the pre-sample estimates as “true infestation”, and consider the performance of smaller samples in estimating infestation for the larger pre-sample.

**Table 1:** Negative-binomial fits for gall counts and for (rounded) percentage of needles galled.

Site	N	Mean gall count	Estimated *k*	?^2^	df	*P*	Mean % galling	Precision of estimate	Estimated *k*	?^2^	df	*P*
A	100	38.2	0.511	152	54	**<10^−10^**	3.80	±23%	0.763	15.9	14	0.32
B	200	43.0	0.888	197	84	**<10^−10^**	4.95	±12%	1.46	22.6	18	0.21
C	200	77.6	1.07	270	115	**<10^−13^**	7.02	±13%	1.44	44.2	26	**0.014**
D	200	54.2	0.773	241	97	**<10^−13^**	4.53	±15%	1.15	33.8	19	**0.020**
E	200	11.0	0.595	85.9	39	**2×10^−5^**	1.04	±17%	1.37	7.35	6	0.29
F	100	11.8	0.686	63.9	32	**7×10^−4^**	1.12	±22%	2.72	4.55	4	0.34
G	200	17.4	0.510	142	50	**<10^−9^**	1.80	±19%	0.73	16.2	12	0.18

Entries for χ^2^, df, and *P* are for likelihood-ratio goodness-of-fits tests. For % galling, “precision” is half the width of the 95% confidence envelope divided by the estimated infestation (see Figure S5).

### Analyzing *P. tumifex* distributions

**Figure 1:**
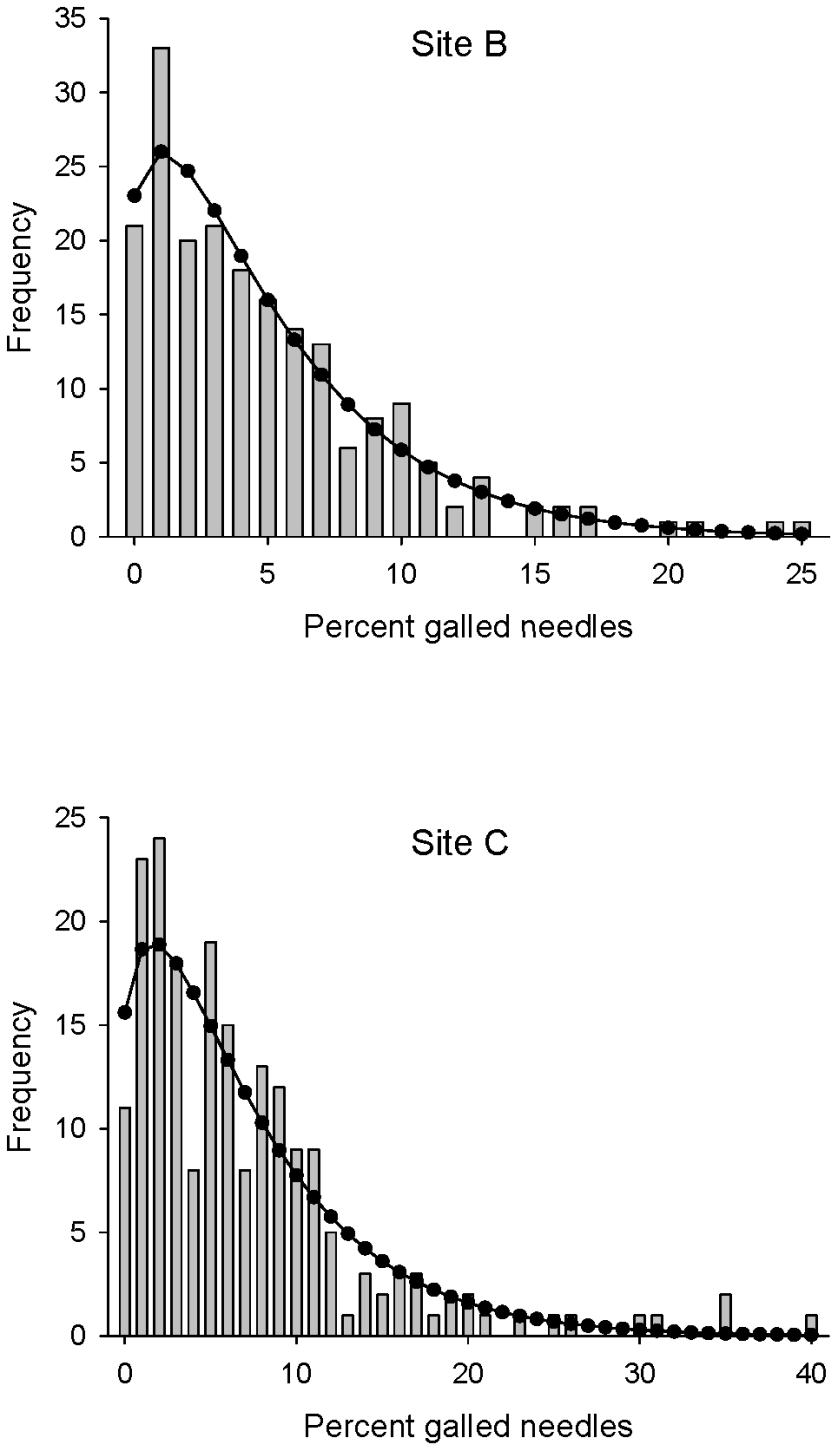
Negative binomial fits for percentage of needles galled, sites B and C. Site B is typical of sites with acceptable fits, whereas Site C is the worst-fitting site. Fits for all seven sites appear in Figure S3.

Both raw gall counts and percentages of galled needles showed highly right-skewed distributions, and neither could be credibly fit to normal distributions (results not shown). Attempts to fit raw gall counts to a negative binomial distribution also failed (Table 1). However, percentages of needles galled (rounded to the nearest 1% for analysis as pseudo-counts) fit negative binomial distributions well (Fig. 1, Fig. S3, Table 1): sites C and D showed significant but modest deviations from the theoretical distribution, whereas all other sites showed excellent fits.

**Figure 2:**
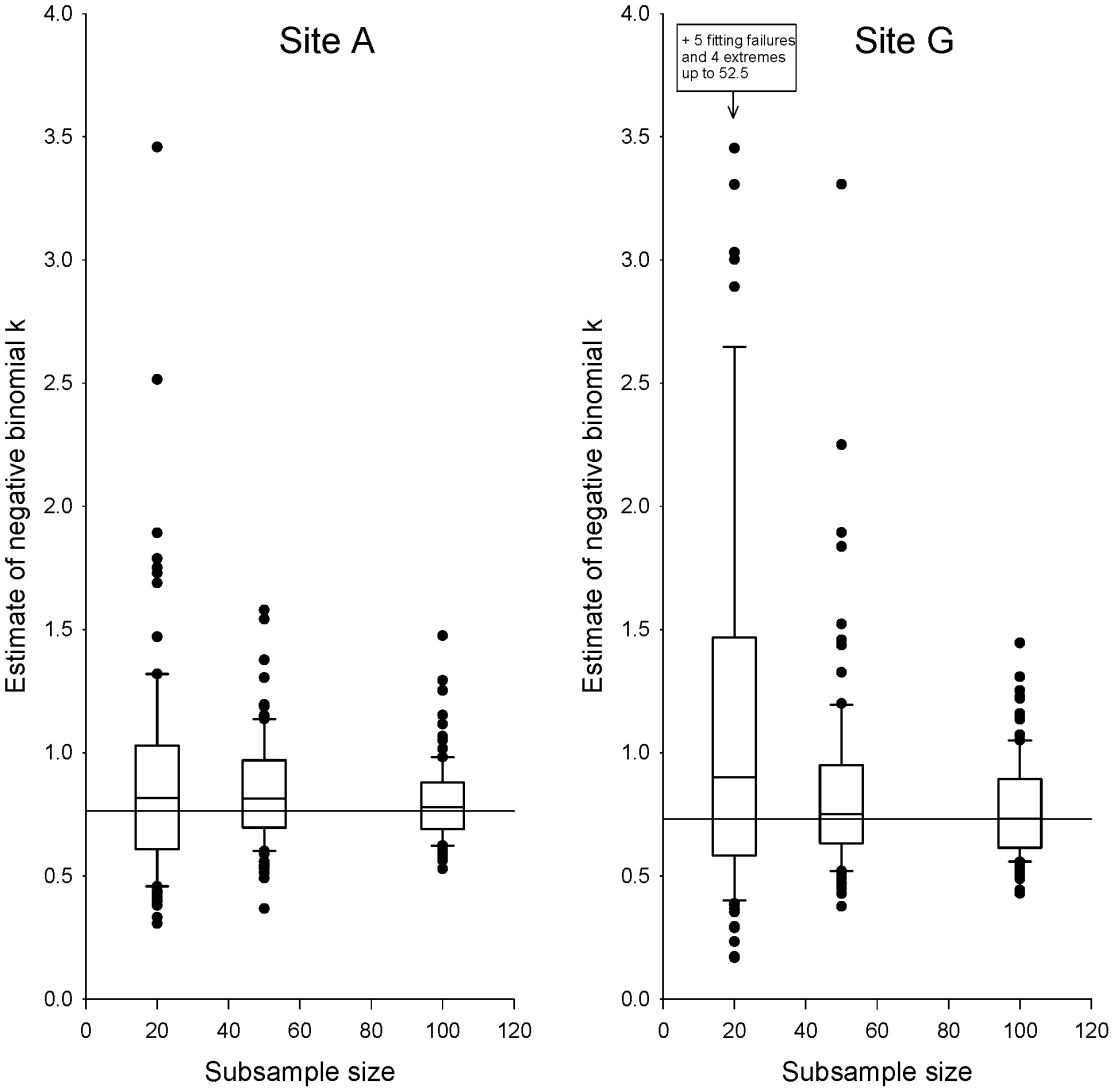
Variation in estimation of negative-binomial *k* at sites A and G, based on subsampling. Horizontal line indicates the true value of *k* (estimated using the full data set). Boxes show central 50%, and whiskers central 90%, of estimates. Plots for all seven sites appear in Figure S4.

Estimates of the negative binomial clumping parameter, *k*, based on reasonably sized subsamples of trees proved very poor. At site A, estimates based on subsamples of *n* = 20 ranged 11-fold, and even subsamples of *n* = 100 produced estimates ranging nearly three-fold (Fig. 2). Estimation performed worse at all other sites (Fig. 2, Fig. S4), and particularly poorly at the lower-density sites E, F, and G. The best of the low-density sites, site G, yielded *k* estimates for subsamples of *n* = 20 that ranged 400-fold. However, for some samples at sites E, F, and G, we were not able to fit negative binomial distributions at all; these samples had to be omitted from our analyses. Therefore, parameter estimation for low-density sites was actually even more difficult than suggested by the results reported here.

**Figure 3:**
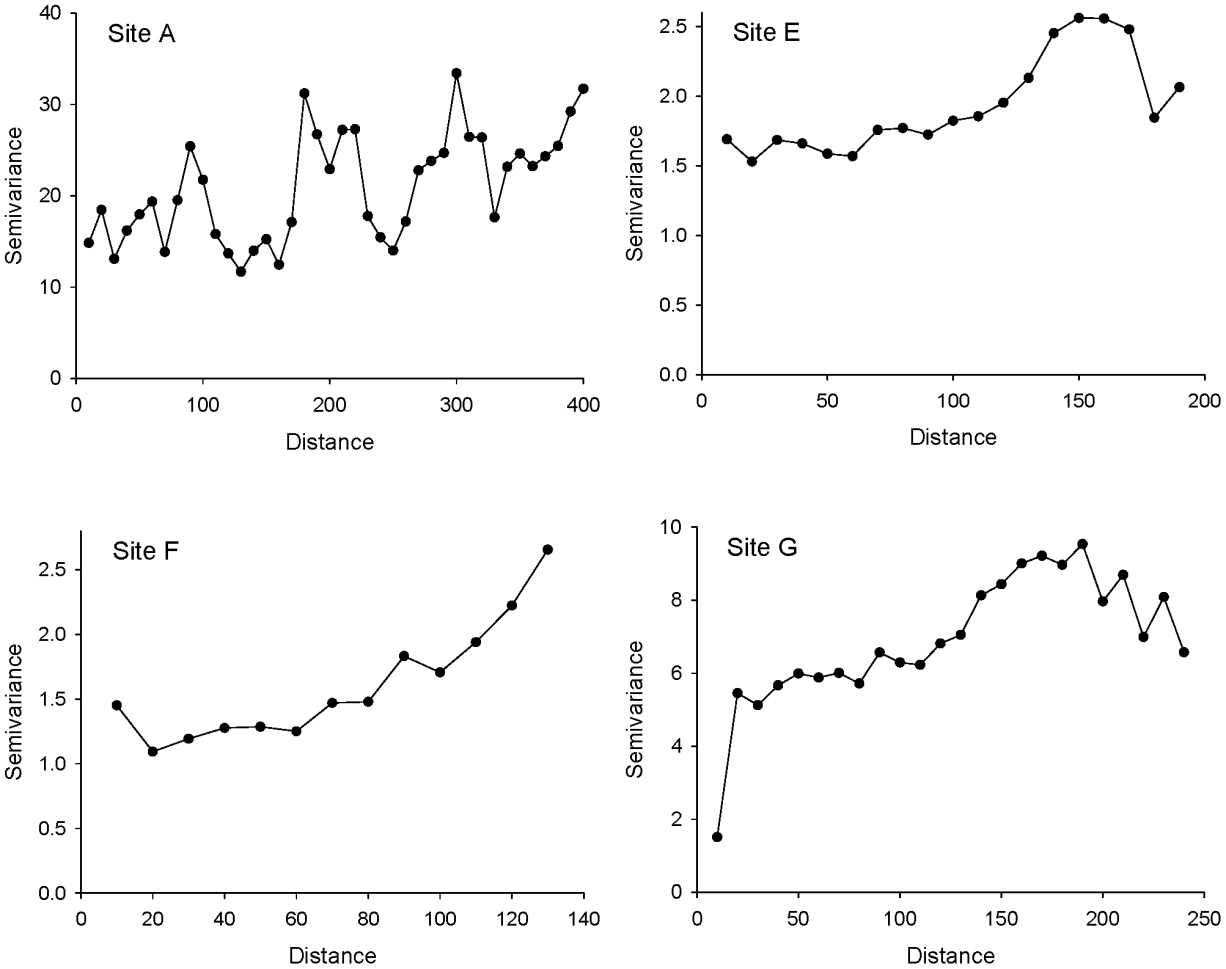
Spatial structure in *P. tumifex* infestation at sites with significant spatial autocorrelation.

We detected significant within-site spatial autocorrelation at four of our seven sites (Table 2), although correlations between geographic and infestation distances were modest (all Mantel *r*<0.13). Spatial autocorrelation remained at relatively large lag distances (Fig. 3), typically on the order of half the longest dimension of the site. There may have been further or stronger autocorrelation on very short spatial scales (<10 m), but our field sampling regime deliberately avoided sampling neighbours at those scales.

**Table 2:** Tests for within-site spatial autocorrelation in *P. tumifex* infestation.

Site	Mantel correlation	*P*
A	**0.125**	**0.0088**
B	0.0389	0.071
C	0.00280	0.45
D	0.0135	0.31
E	**0.0683**	**0.0096**
F	**0.113**	**0.0055**
G	**0.0709**	**0.026**

### Simulated sampling

**Figure 4:**
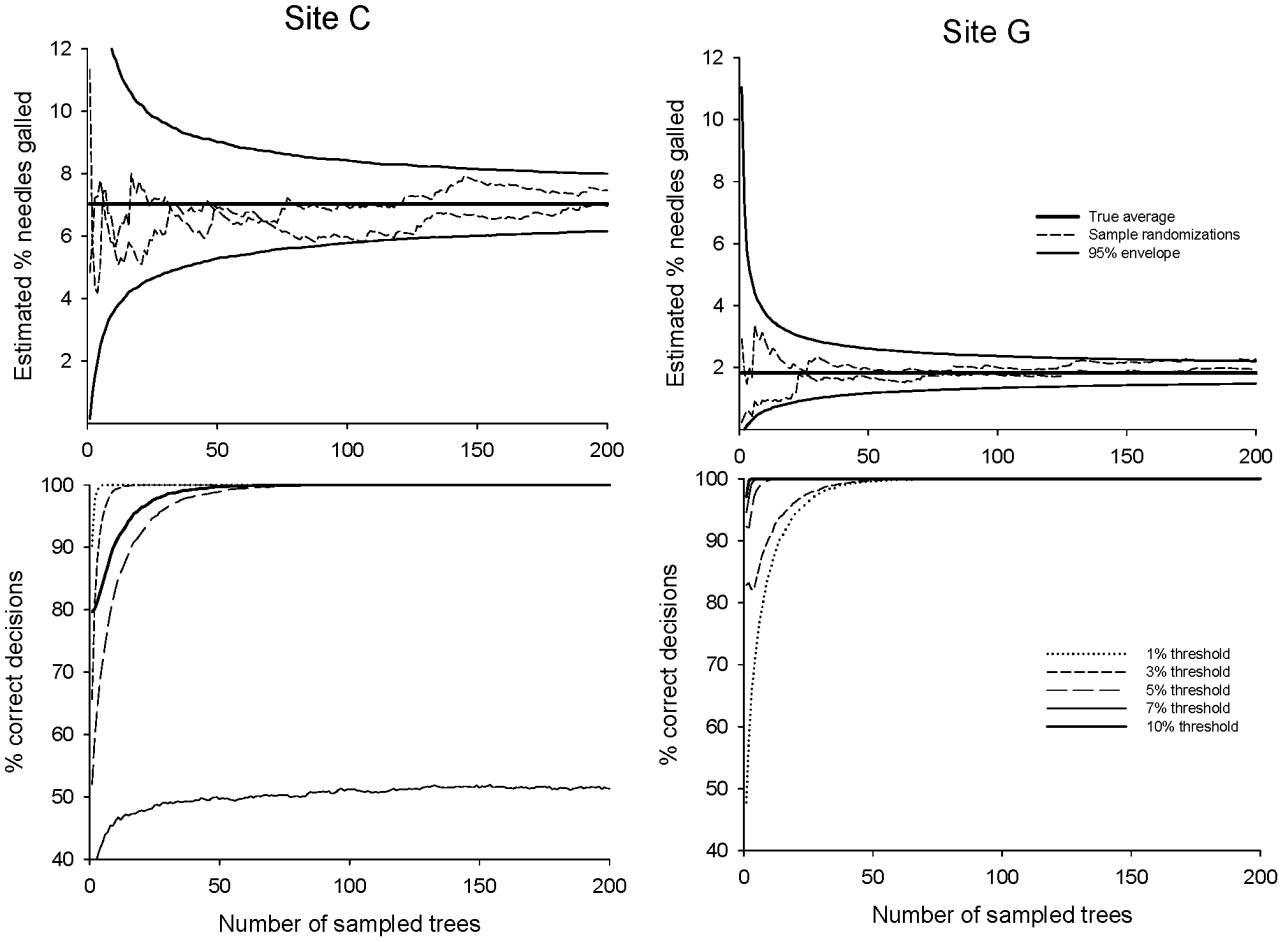
Performance of random sampling for estimating mean *P. tumifex* density (top panels) and decision making against infestation thresholds (bottom panels) at representative sites C and G. Dashed lines show two representative randomizations; 95% of the 10,000 randomizations lie between the solid lines. Confidence envelopes still have finite width at *n* = 200 (the size of the total site sample) because sampling is conducted with replacement. Plots for all seven sites appear in Figure S5.

Random sampling produced, as expected, infestation estimates that approached the true mean with increasing *n* (Fig. 4, top panels). For all sites, the confidence intervals narrow rapidly, and 95% certainty of estimation to within ±50% of the true infestation rate is possible for *n* = 25 or smaller. When the goal is inference about infestation relative to a threshold, random sampling allowed correct decision rates in excess of 90% for quite small *n*, on the order of 25–40 trees (Fig. 4, bottom panels) with very few exceptions. Making correct decisions is, of course, difficult when the decision threshold is very close to the true infestation rate: for example, for the 7% threshold at site C (true infestation, 7.02%).

**Figure 5:**
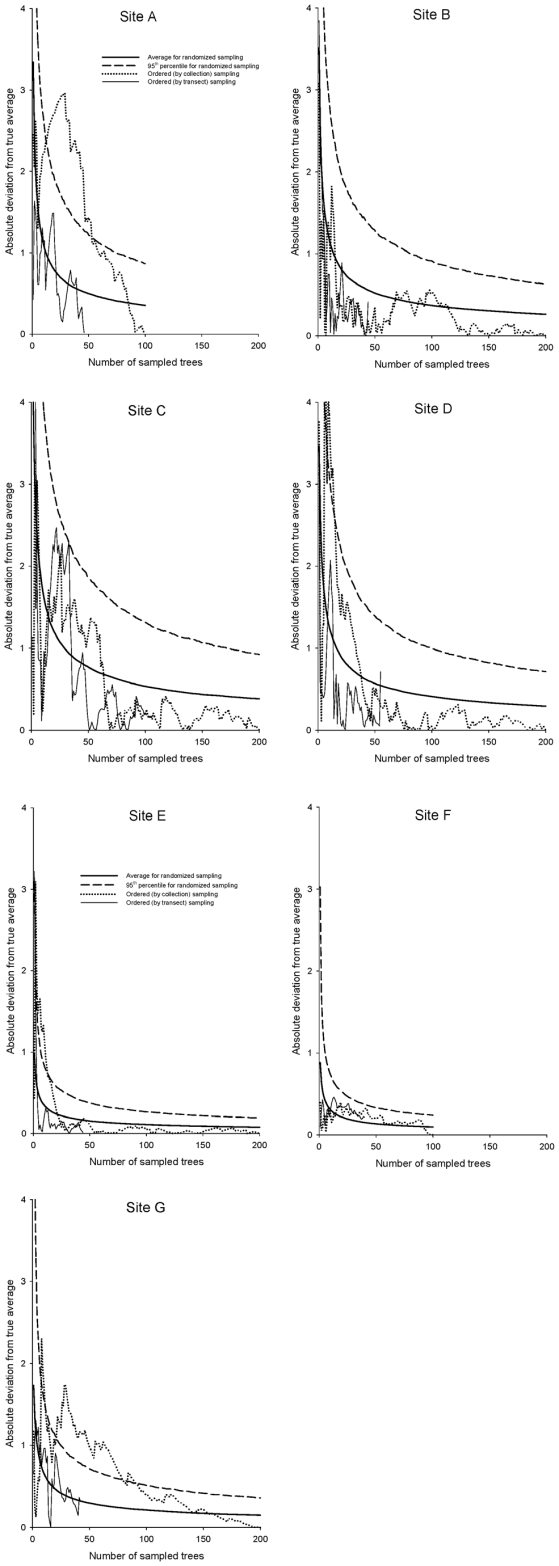
Estimation errors for *P. tumifex* infestation as a function of number of sampled trees.

Expected errors in infestation estimates for random sampling decrease rapidly with *n* (Fig. 5, heavy solid and dashed lines). Interestingly, sampling ordered by collection number performed comparably to random sampling (Fig. 5, dotted lines): with the exception of site A, estimation error was usually below the 95^th^ percentile for random sampling and very often below the average for random sampling. Transect sampling performed even better (Fig. 5, light solid lines): estimation error never exceeded the 95^th^ percentile for random sampling and was below the average for random sampling more often than above, even for small *n*. Reversing the direction of sampling by collection number or along transects produced results that differed in detail but not in overall interpretation (results not shown).

## Discussion

### Implications for *P. tumifex* biology

Our sampling data indicate that both within- and between-site distributions of *P. tumifex* are complex. Within sites, we were not able to fit raw gall counts to any simple distribution, most likely because the shoots we sampled varied in size, and *P. tumifex* attack is influenced by shoot length [2]. Rather than exploring distributions with additional parameters to allow direct modeling of this dependence, we calculated infestation as the percentage of galled needles, which is a measure of direct importance to host trees and to farmers. After rounding, this measure was well described by negative binomial distributions (that is, infestation shows noticeable clumpiness across trees). However, we found nearly four-fold variation among sites in estimated values of the clumping parameter *k*. In other words, *P. tumifex* distributions *within* sites, and therefore patterns in *P. tumifex* damage, vary significantly *among* sites. The mechanisms underlying this variation may be difficult to identify in the context of Christmas tree farms given great variation among farms in size, shape, landscape context, and so on. We are currently examining the ecology of *P. tumifex*'s movement and activity patterns in an attempt to understand within-stand dispersal and how it shapes spatial and temporal patterns in damage to the host trees.

### Sampling strategies for *P. tumifex*

Our *P. tumifex* data proved quite recalcitrant to the application of parametric sequential sampling. Although we were able to fit rounded percentage galling data to the negative binomial distribution, the strong variation in *k* among sites means that single-parameterization sequential sampling would be misleading for *P. tumifex*. Local-parameterization methods could accommodate variation in *k*, but we found that very large warm-up samples would be needed for accurate estimation of local *k*, defeating the efficient-sampling purpose of sequential sampling. That we encountered this problem is not surprising, given the difficulty of estimating *k* for negative binomial distributions (especially when the mean is small; [20]–[22]. To make the situation even worse, the presence of modest but significant spatial autocorrelation at several of our sites should cast doubt on even local-parameterization strategies for *P. tumifex* [19].

Fortunately, our simulations showed that simpler approaches to sampling provide adequate density estimates for *P. tumifex* without requiring large sample sizes. In fact, adequate estimation was possible at all our sites with samples considerably smaller than the warm-up samples that would have been needed for local-parameterization sequential sampling. Furthermore, we were able to evaluate efficiency for alternative ways of selecting trees to be sampled. We found that sampling trees in the convenient order used by field crews was about as good as random sampling, and sampling trees via belt transects was actually better. This is good news, because random sampling can be cumbersome to implement in the field and therefore carries additional costs. Belt transects offer relatively easy fieldwork, and probably perform well at density estimation because they cut across the kind of spatial variation in attack revealed by our autocorrelation analyses.

We can offer a very simple recommendation for Christmas tree farmers in Atlantic Canada. *Paradiplosis tumifex* infestation can be assessed to reasonable accuracy by sampling trees in 10 m wide belt transects, placed along a major axis of the farm, and long enough to include 25–40 trees spaced at least 10 m apart. (In our farms, this was enough trees for the transect to span most or all of the long axis of the farm. In a larger farm, it would seem sensible either to include more trees, or more likely, to assess infestation and deploy intervention separately for two or more plots within the farm.) Where decision making against thresholds is desired, similar sample sizes provide excellent accuracy except where estimated densities are very close to the decision threshold. This situation is easily recognized and can be dealt with by deciding on intervention at estimated infestations slightly lower than the true threshold. This recommended approach was robust to the considerable variation in stand characteristics and agricultural practices across our seven study farms. More sophisticated sampling schemes that formalize estimation by fully parameterizing *P. tumifex* distributions would require substantially more effort, while returning little improvement in results.

### Lessons and tools for assessing sampling strategies

There is an enormous amount of literature on the design of sampling strategies for the estimation of population densities in nature. Its existence is good evidence that sampling well is difficult - and being confident that you are sampling well is no less so. Although more accurate estimates usually come from larger sample sizes and more sophisticated sampling, this rule is not inescapable. As a result, tools that allow practitioners to increase efficiency and to assess sampling strategies in advance of large-scale field work will always be valuable.

Our work with *P. tumifex* demonstrates one valuable approach to sampling design. By investing in some pre-sampling and using simulation techniques, we were able to assess the performance of alternative sampling strategies. For *P. tumifex*, sequential sampling was outperformed by simpler approaches that nonetheless appear robust to among-site variation in insect distribution. Sequential sampling is appealing because it promises very high sampling efficiency, but it demands that the statistical distribution from which infestation rates are sampled have parameters that are either known or easily estimated. For *P. tumifex*, these demands were not met, and therefore sequential sampling would have required increased, not decreased, investment in sampling effort. We suspect that *P. tumifex* is not exceptional in this regard.

Of course, the specific sampling scheme we recommend for *P. tumifex* in Atlantic Canada may not perform well in other systems. However, the methods we illustrate and the new software tools we provide can easily be applied to other systems. Implementing our approach requires only a pre-sampling dataset of reasonable size. This dataset should include infestation data for *n* sampling units at each of *s* sites. Both the unit for measuring infestation (% attack, number of insects, etc.) and the identity of the sampling unit (plant, quadrat, etc.) can be chosen as appropriate for the particular system. The dataset should also include (x, y) co-ordinate data for each sampling unit (lacking such data, random sampling can be evaluated but not transect or ordered sampling). The pre-sample size *n* should be large enough that plots like Figure 4 either attain good sampling performance or reach feasibility limits for ongoing sampling. The number of sites *s* should be large enough to be reasonably representative of sites for which ongoing sampling might be desired. A sample dataset (for one site) is included as Dataset S1, and the sequence of analyses is summarized in Figure S6. Following this sequence should make it straightforward for practitioners to establish whether, in any given system, sequential sampling can be applied via single or local parameterization, whether simpler sampling regimes can provide more efficient estimation, and what spatial approach to the selection of sampling units should be preferred. A useful direction for future research would be to extend our software to other candidate sampling schemes, such as stratified sampling.

Pre-sampling and simulation methods offer the chance to compare and optimize potential sampling strategies before they are brought to real-world applications. Our method is not the first of this type. For example, geostatistical analyses have been used to identify optimum sample sizes for pheromone trap monitoring [7], and software is available for assessing distance sampling designs [6]. More generally, the development of simulation-based methods for assessing sampling strategies mirrors the burgeoning use of simulation and randomization-based methods in inferential statistics (e.g., [29], [30]), phylogenetic inference (e.g., [31], [32]), macroevolution (e.g., [33], [34]), and many other fields. In all these applications, the availability of computational power has allowed the relaxation of restrictive assumptions necessary for older parametric approaches – often leading to gains in the efficiency of data use, as we found for sampling *P. tumifex*.

## Supporting Information

Figure S1 **Site maps.** Open circles denote trees included in the full site samples; solid dots mark trees included in sampling by belt transects. Lines mark centres of the first belt transects in each transect set, and arrows mark transect start points.
(PDF)

Figure S2 **Sampling schemes, illustrated for Site G.** (A) Transect sampling (line indicates first transect; filled dots to each side are the jittered transects). (B) Random sampling; dots show first 25 (and arrow first 5) trees chosen in an arbitrary randomization. (C) Ordered sampling (first 20 trees chosen; raster continues for larger samples).
(PDF)

Figure S3 **Negative binomial fits for (rounded) percent needles galled, for all sites.**
(PDF)

Figure S4 **Estimation of negative-binomial** ***k*** **for all sites.** Horizontal line indicates the true value of *k* (estimated using the full dataset). Boxes show central 50%, and whiskers central 90%, of estimates.
(PDF)

Figure S5 **Performance of random sampling for estimating mean** ***P. tumifex*** **density (top panels) and decision-making against infestation thresholds (bottom panels) at all sites.** Dashed lines show two representative randomizations; 95% of the 10,000 randomizations lie between the solid lines. Confidence envelopes still have finite width at *n* = 200 (the size of the total site sample) because sampling is conducted with replacement.
(PDF)

Figure S6 **Summary of our analytical approach.** Italics refer to R scripts and software available as Software S1, S2, S3, S4.
(PDF)

Dataset S1 **Sample dataset (for our site G).**
(CSV)

Software S1 **R scripts used in our analysis.**
(TXT)

Software S2 **Zipped executable version of software InfestSample version 1.10.**
(ZIP)

Software S3 **Text-file source code of software InfestSample version 1.10.**
(TXT)

Software S4 **Zipped version of Visual Studio project folder for software InfestSample version 1.10.**
(ZIP)
