# Supplementary material for: A Simulation Approach to Assessing Sampling Strategies for Insect Pests: An Example with the Balsam Gall Midge
Source: PLoS One. 2013 Dec 23;8(12):e82618. doi: 10.1371/journal.pone.0082618 (PMC3871163; doi:10.1371/journal.pone.0082618)
Supplement: Figure S1 — Site maps. Open circles denote trees included in the full site samples; solid dots mark trees included in sampling by belt transects. Lines mark centres of the first belt transects in each transect set, and arrows mark transect start points. (PDF) [file pone.0082618.s001.pdf]

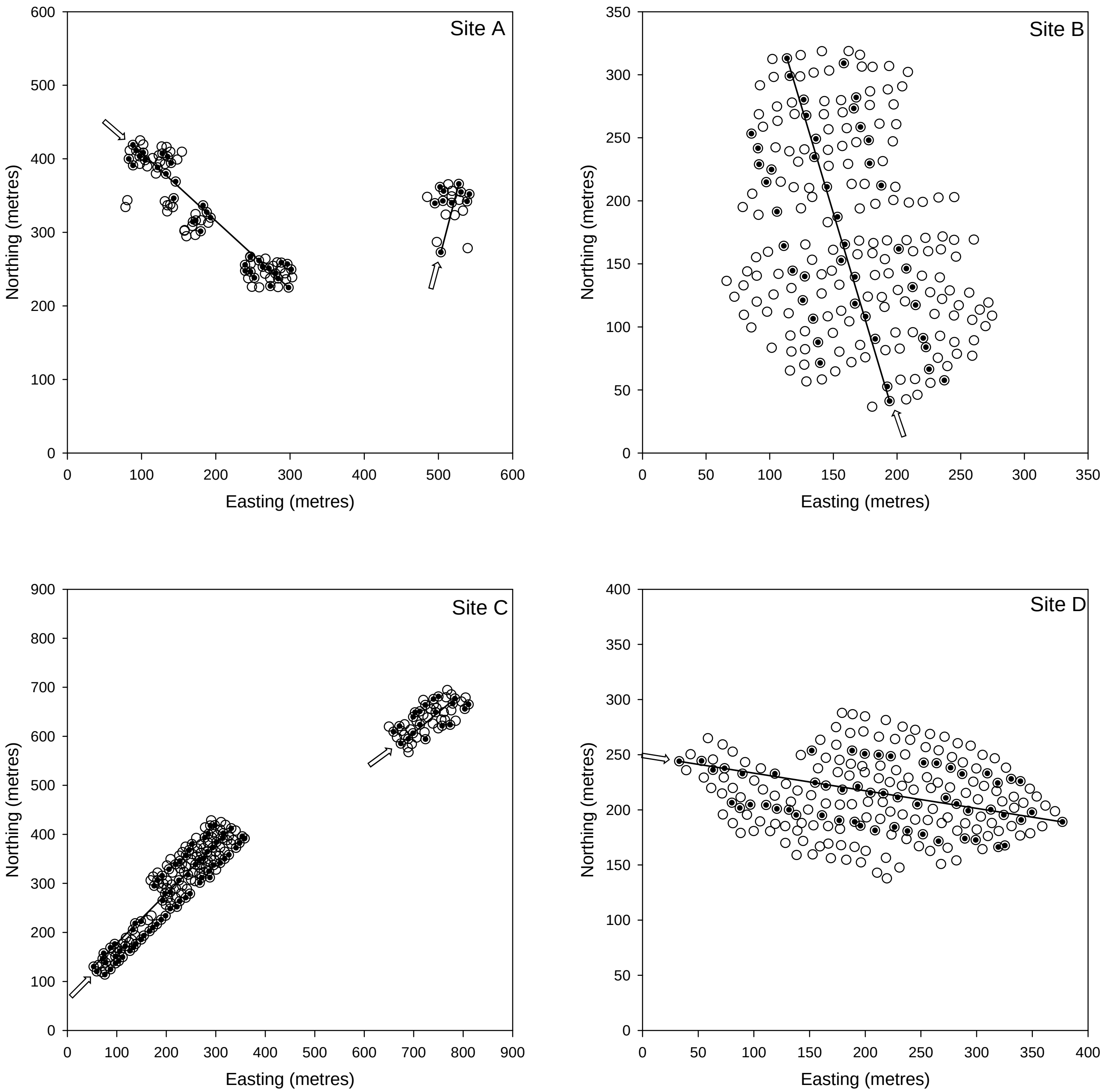

Figure S1. Site maps. Open circles denote trees included in the full site samples; solid dots mark trees included in sampling by belt transects. Lines mark centres of the first belt transects in each transect set, and arrows mark transect start points.

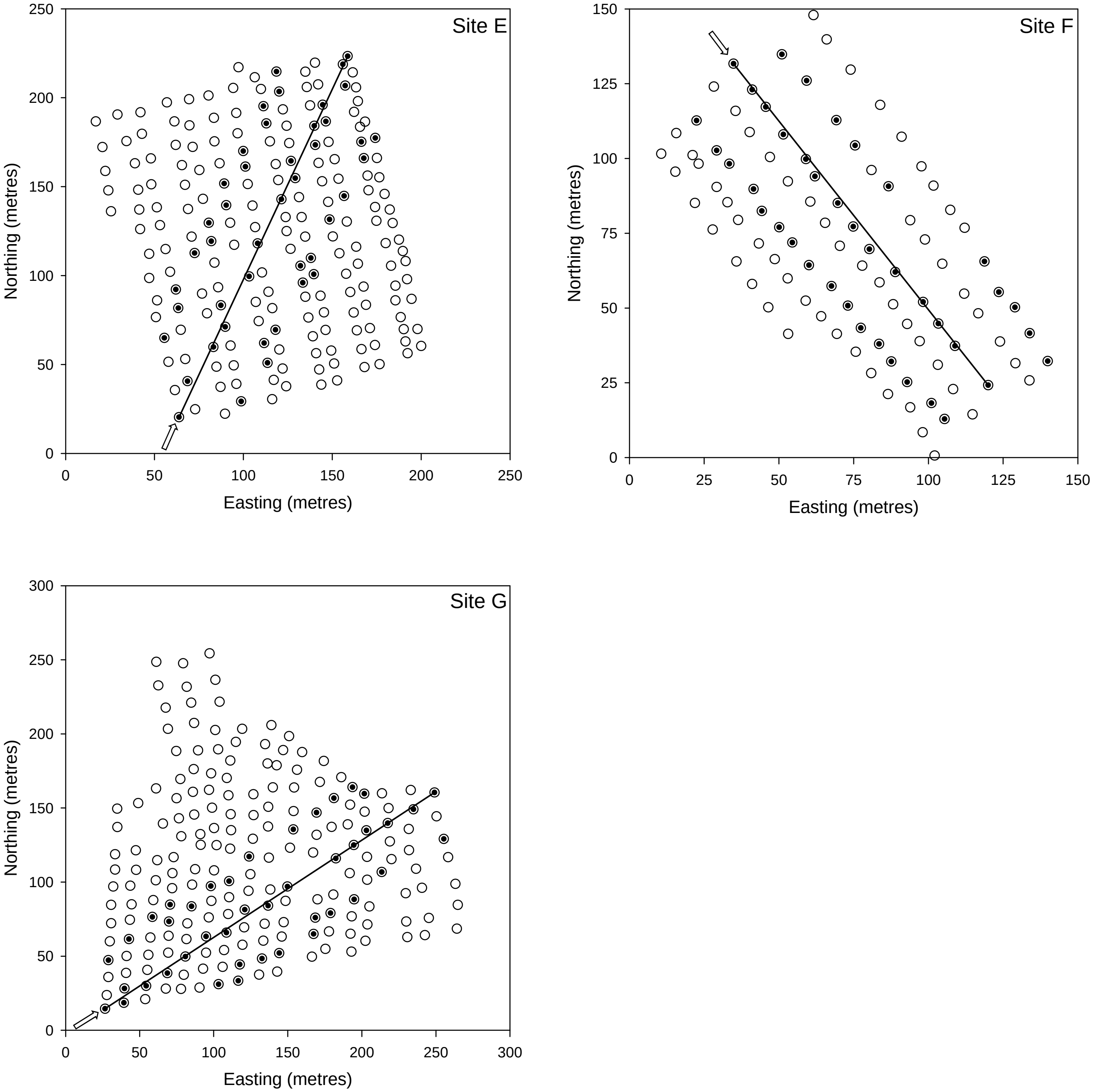

Figure S1. Site maps (continued).
