# Supplementary material for: A Simulation Approach to Assessing Sampling Strategies for Insect Pests: An Example with the Balsam Gall Midge
Source: PLoS One. 2013 Dec 23;8(12):e82618. doi: 10.1371/journal.pone.0082618 (PMC3871163; doi:10.1371/journal.pone.0082618)
Supplement: Figure S2 — Sampling schemes, illustrated for Site G. (A) Transect sampling (line indicates first transect; filled dots to each side are the jittered transects). (B) Random sampling; dots show first 25 (and arrow first 5) trees chosen in an arbitrary randomization. (C) Ordered sampling (first 20 trees chosen; raster continues for larger samples). (PDF) [file pone.0082618.s002.pdf]

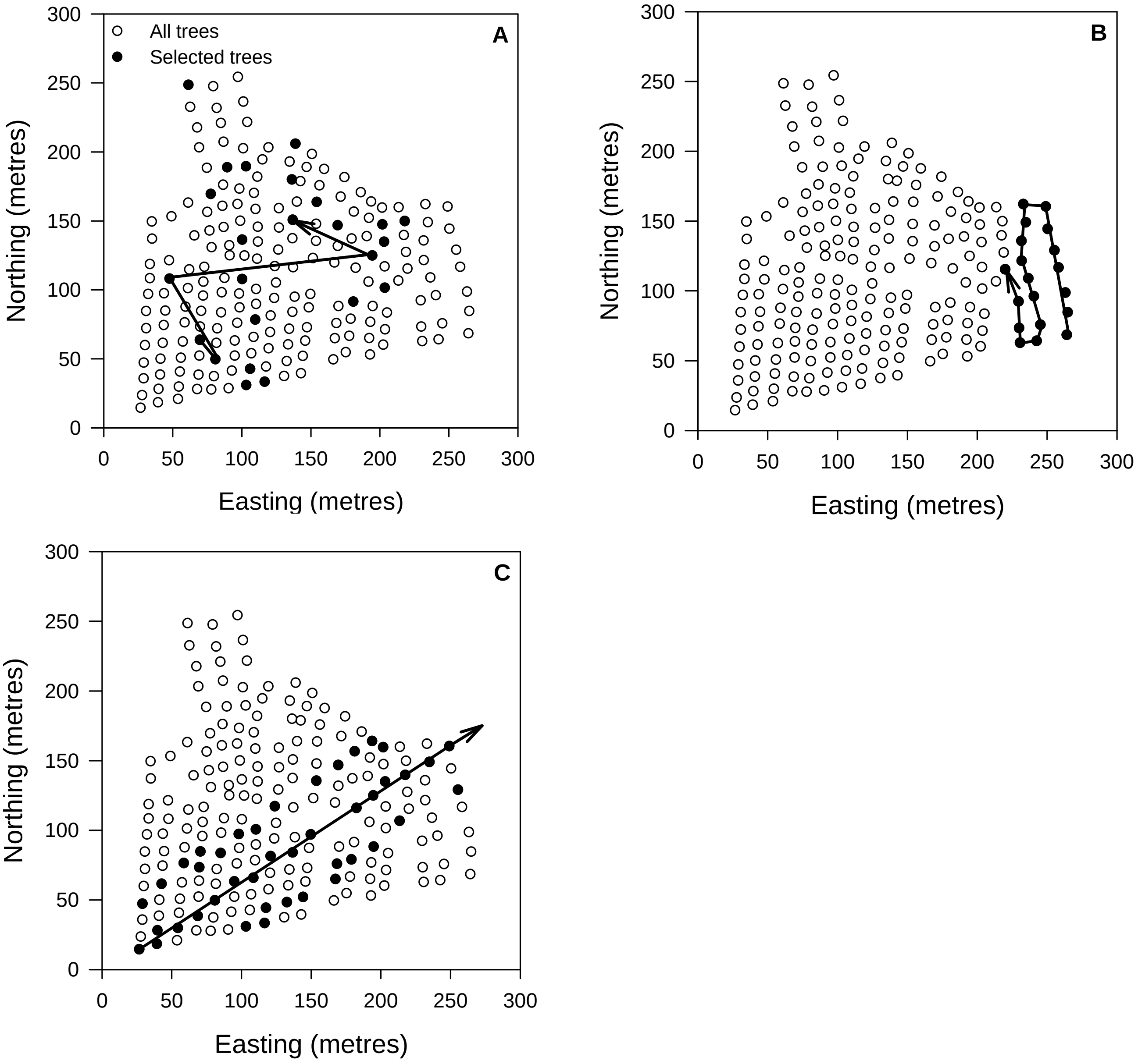

Figure S2. Sampling schemes, illustrated for Site G. (A) Transect sampling (line indicates first transect; filled dots to each side are the jittered transects). (B) Random sampling; dots show first 25 (and arrow first 5) trees chosen in an arbitrary randomization. (C) Ordered sampling (first 20 trees chosen; raster continues for larger samples).
