# Supplementary material for: A Simulation Approach to Assessing Sampling Strategies for Insect Pests: An Example with the Balsam Gall Midge
Source: PLoS One. 2013 Dec 23;8(12):e82618. doi: 10.1371/journal.pone.0082618 (PMC3871163; doi:10.1371/journal.pone.0082618)
Supplement: Figure S3 — Negative binomial fits for (rounded) percent needles galled, for all sites. (PDF) [file pone.0082618.s003.pdf]

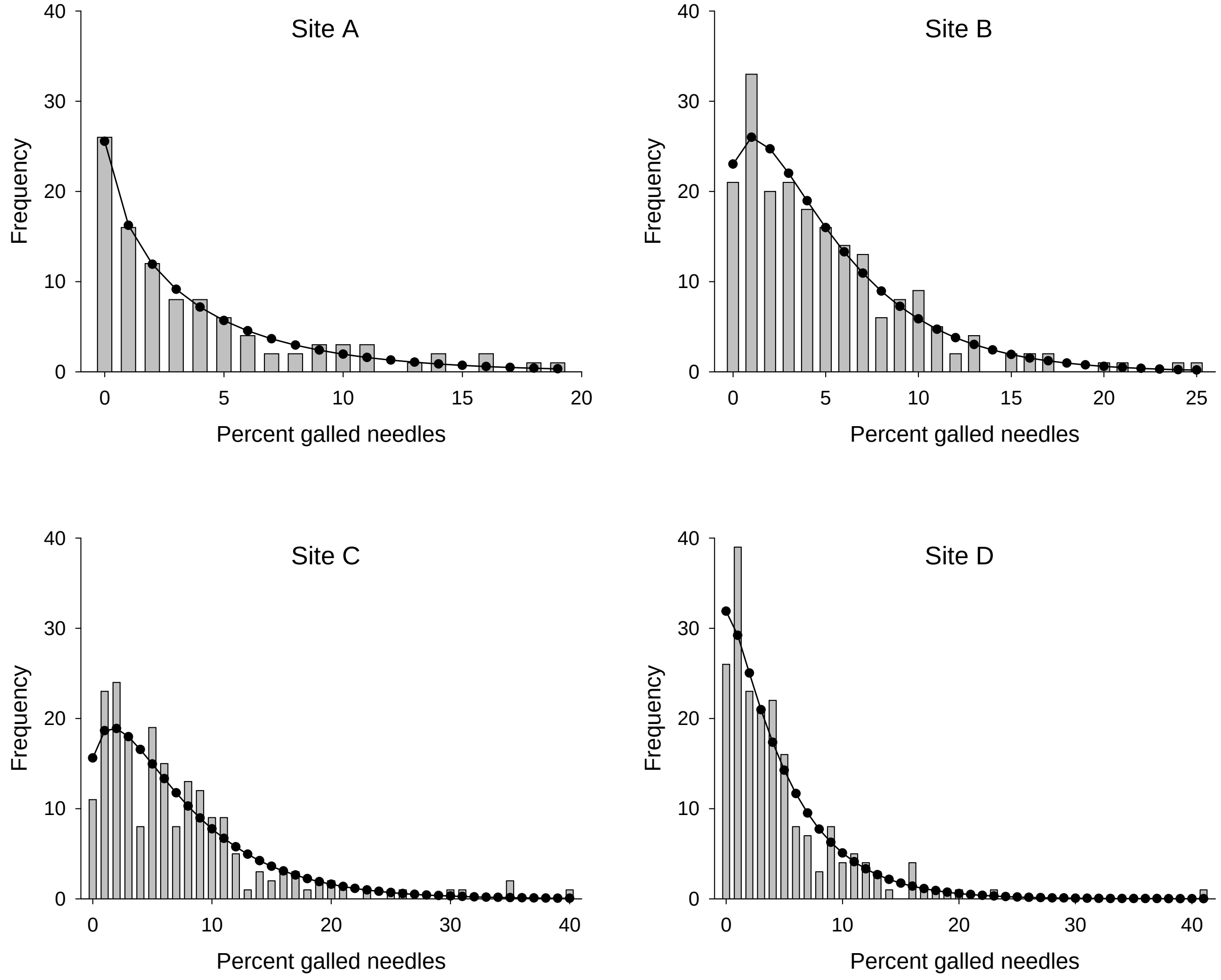

Figure S3. Negative binomial fits for (rounded) percent needles galled, for all sites.

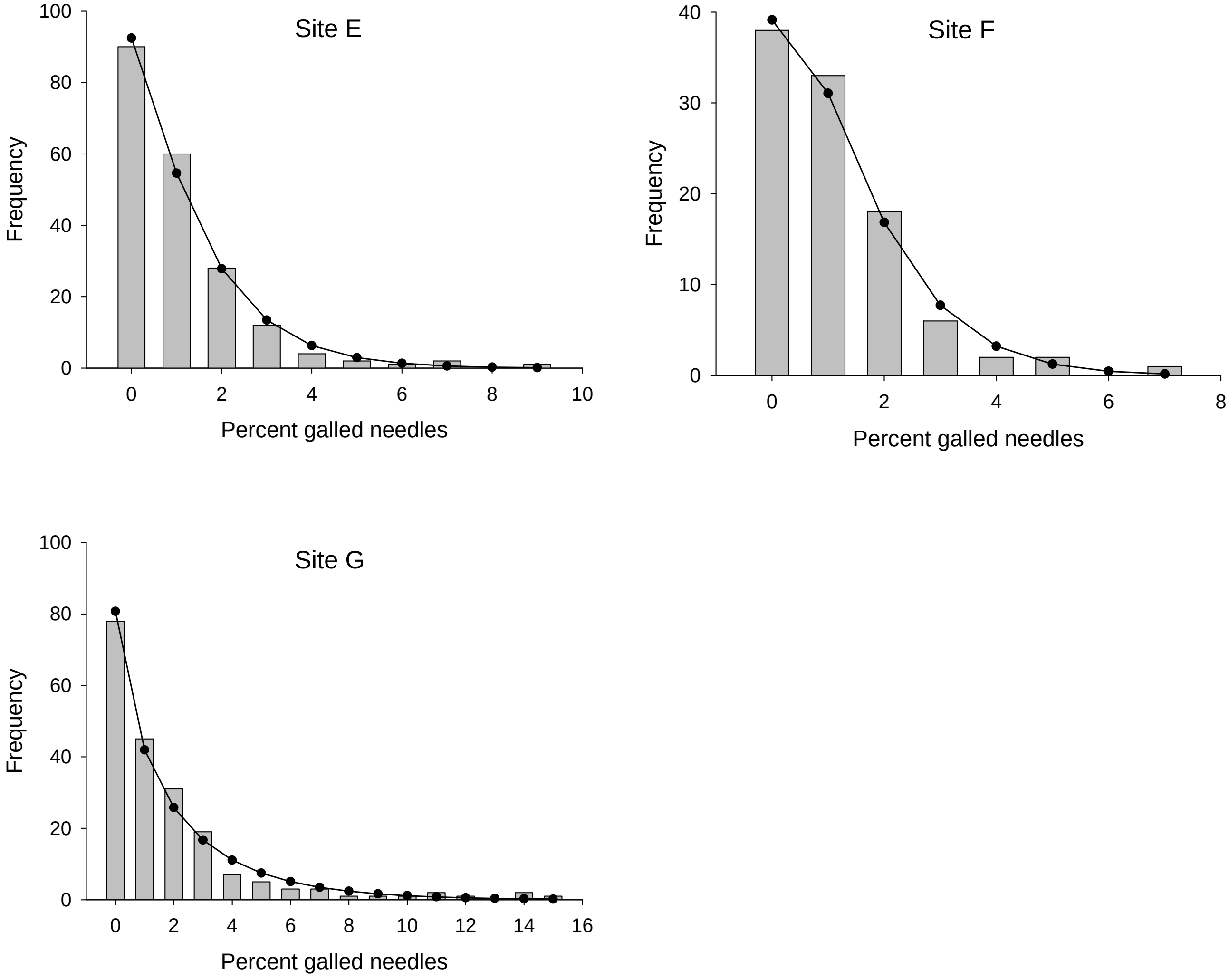

Figure S3. Negative binomial fits (continued).
