# Supplementary material for: A Simulation Approach to Assessing Sampling Strategies for Insect Pests: An Example with the Balsam Gall Midge
Source: PLoS One. 2013 Dec 23;8(12):e82618. doi: 10.1371/journal.pone.0082618 (PMC3871163; doi:10.1371/journal.pone.0082618)
Supplement: Figure S4 — Estimation of negative-binomial k for all sites. Horizontal line indicates the true value of k (estimated using the full dataset). Boxes show central 50%, and whiskers central 90%, of estimates. (PDF) [file pone.0082618.s004.pdf]

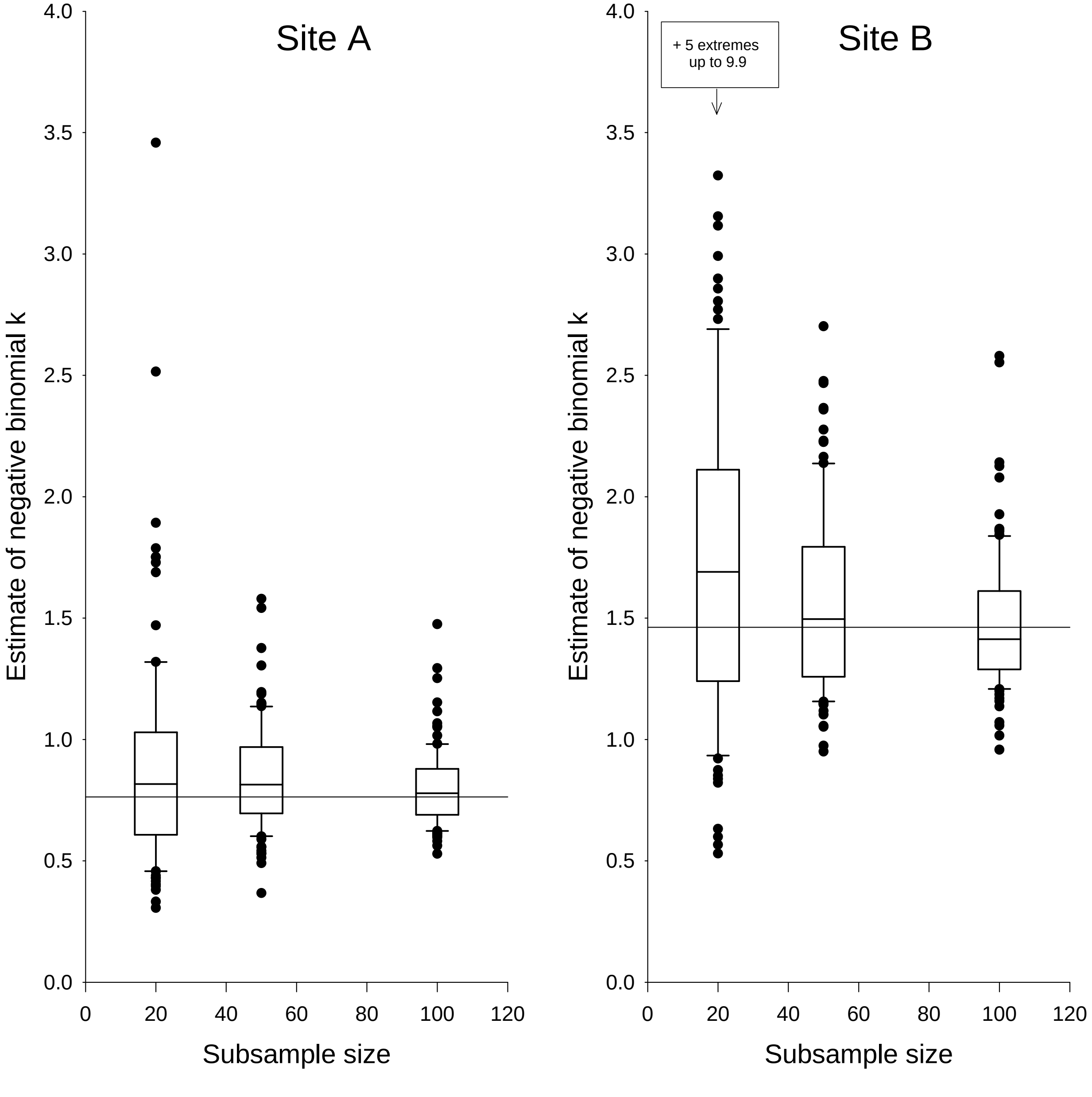

Figure S4. Estimation of negative-binomial *k* for all sites. Horizontal line indicates the true value of *k* (estimated using the full dataset). Boxes show central 50%, and whiskers central 90%, of estimates.

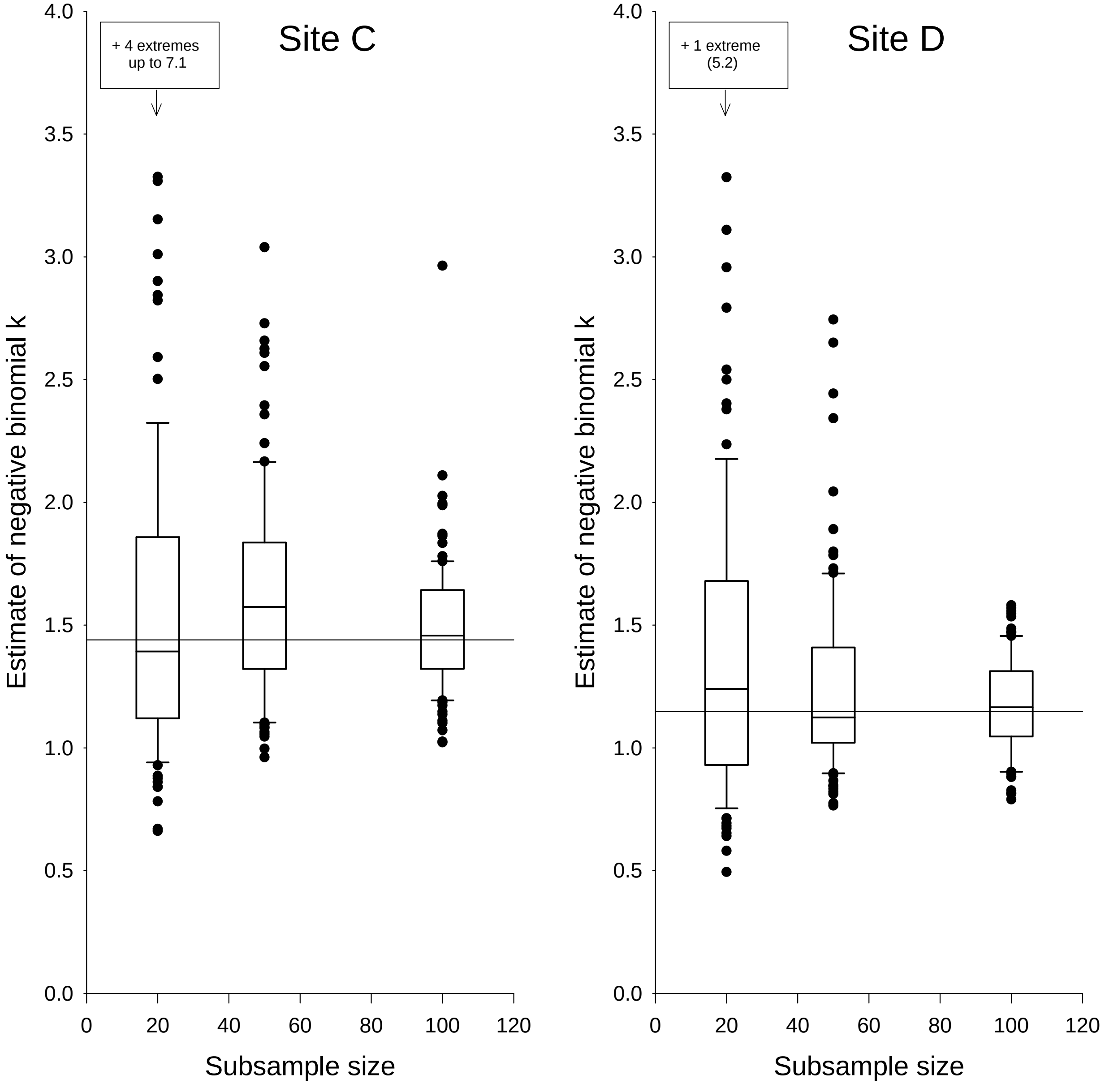

Figure S4. Estimation of negative-binomial *k* (continued).

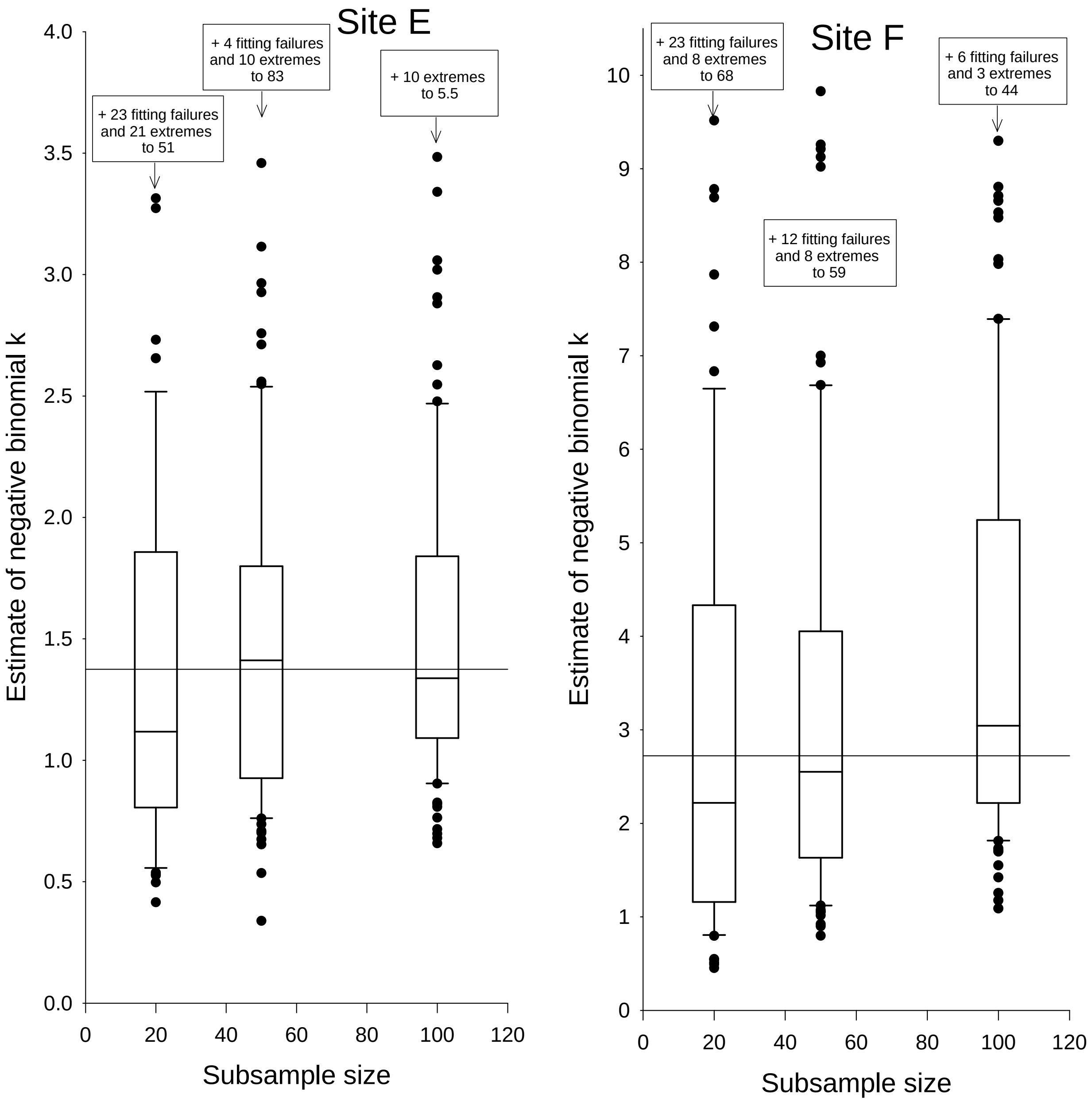

Figure S4. Estimation of negative-binomial *k* (continued).

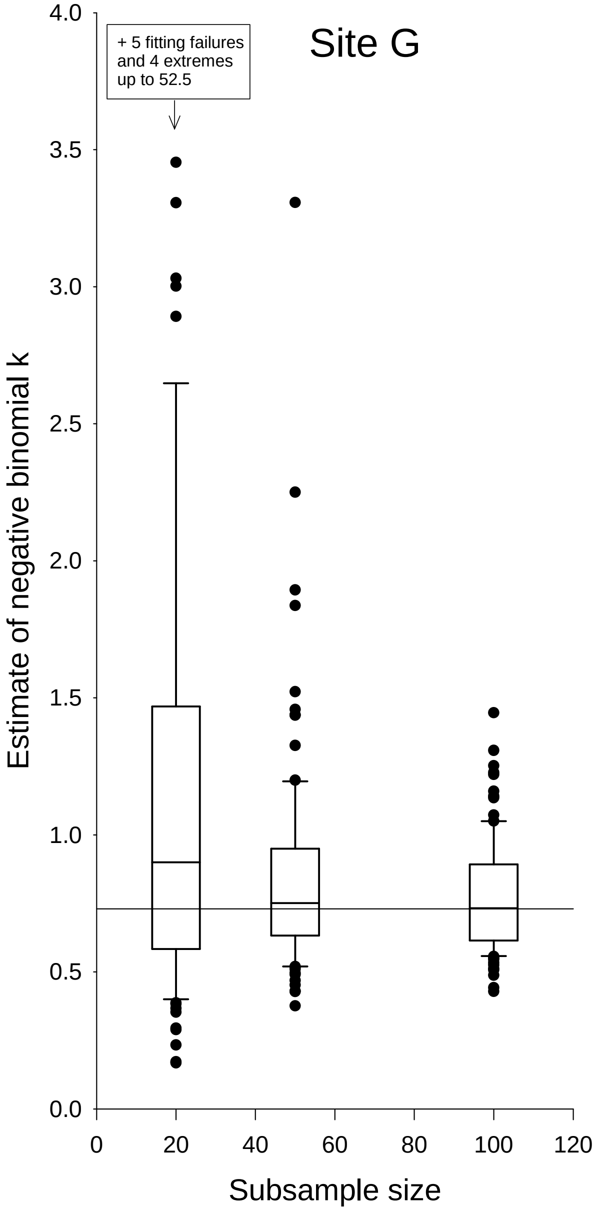

Figure S4. Estimation of negative-binomial *k* (continued).
