# Supplementary material for: A Simulation Approach to Assessing Sampling Strategies for Insect Pests: An Example with the Balsam Gall Midge
Source: PLoS One. 2013 Dec 23;8(12):e82618. doi: 10.1371/journal.pone.0082618 (PMC3871163; doi:10.1371/journal.pone.0082618)
Supplement: Figure S5 — Performance of random sampling for estimating mean P. tumifex density (top panels) and decision-making against infestation thresholds (bottom panels) at all sites. Dashed lines show two representative randomizations; 95% of the 10,000 randomizations lie between the solid lines. Confidence envelopes still have finite width at n = 200 (the size of the total site sample) because sampling is conducted with replacement. (PDF) [file pone.0082618.s005.pdf]

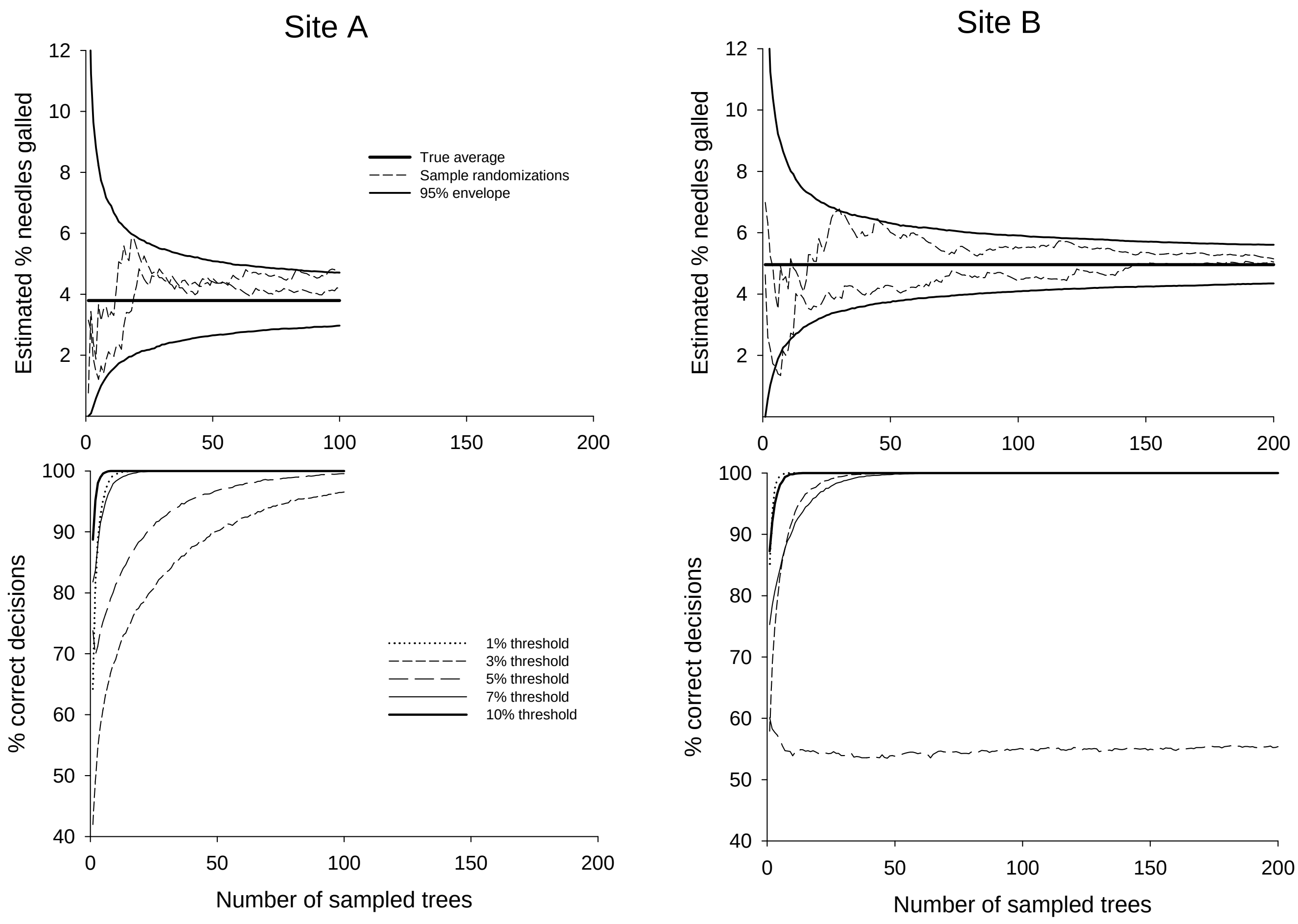

Figure S5. Performance of random sampling for estimating mean *P. tumifex* density (top panels) and decision-making against infestation thresholds (bottom panels) at all sites. Dashed lines show two representative randomizations; 95% of the 10,000 randomizations lie between the solid lines. Confidence envelopes still have finite width at n = 200 (the size of the total site sample) because sampling is conducted with replacement.

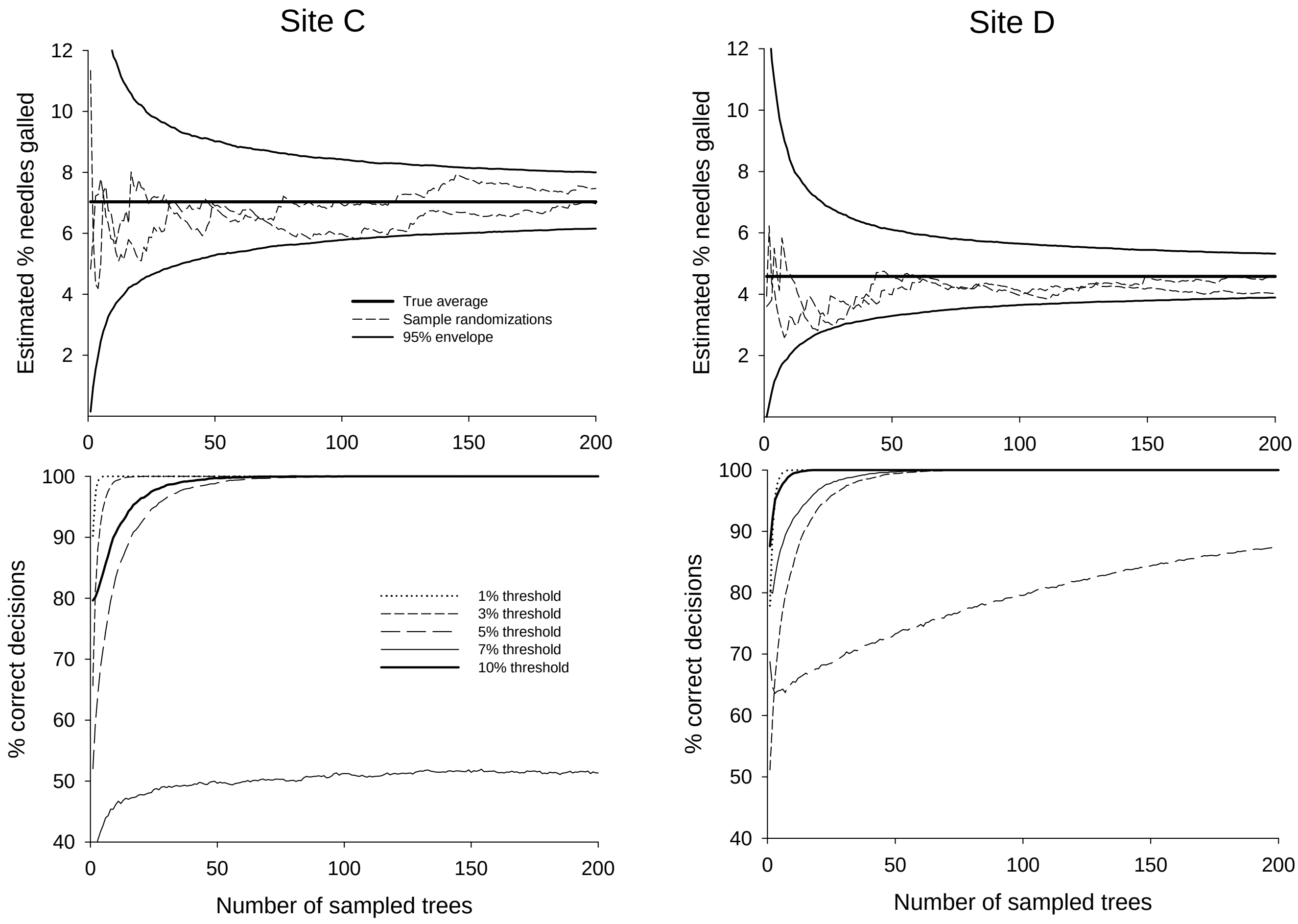

Figure S5. Performance of random sampling (continued).

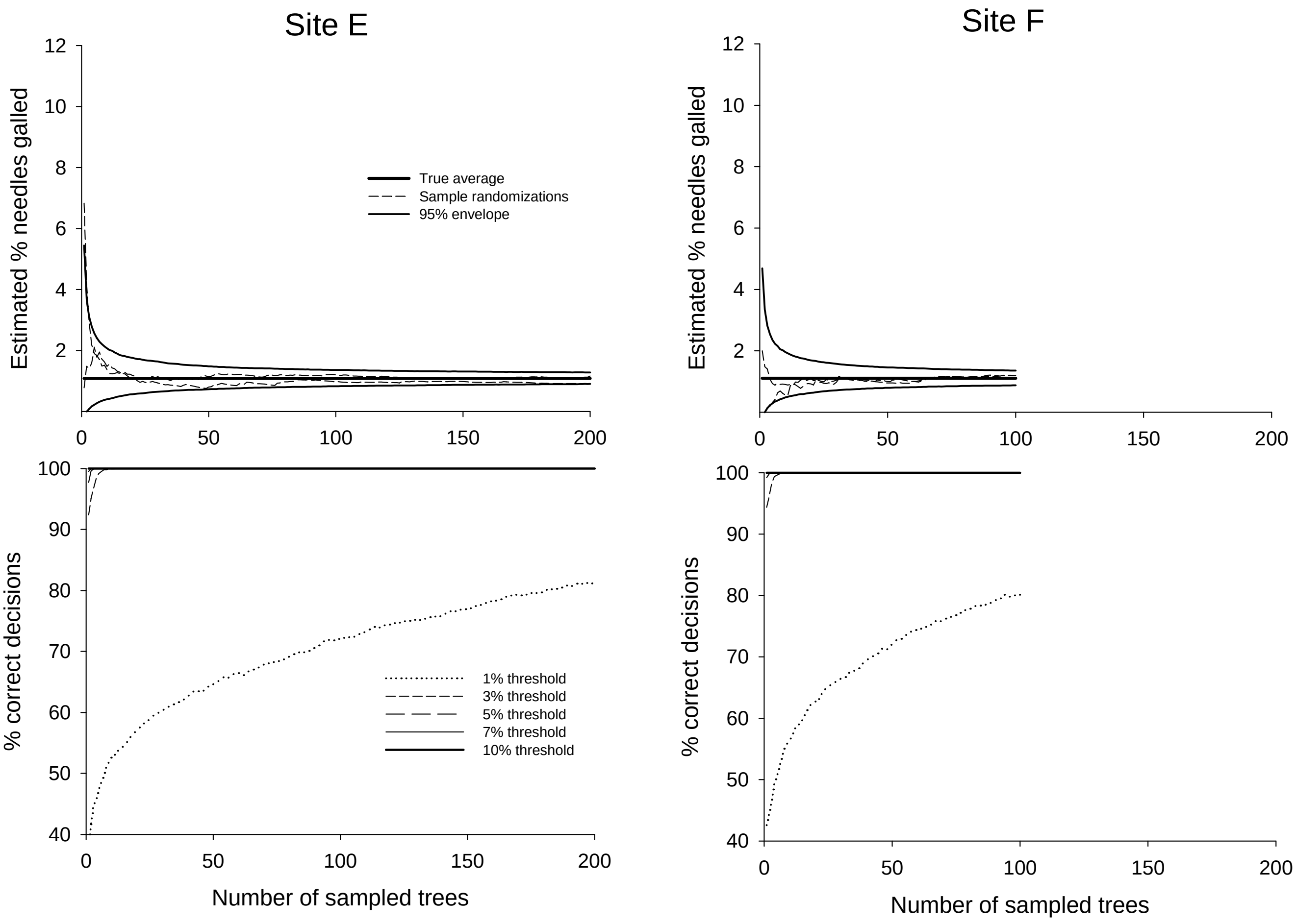

Figure S5. Performance of random sampling (continued).

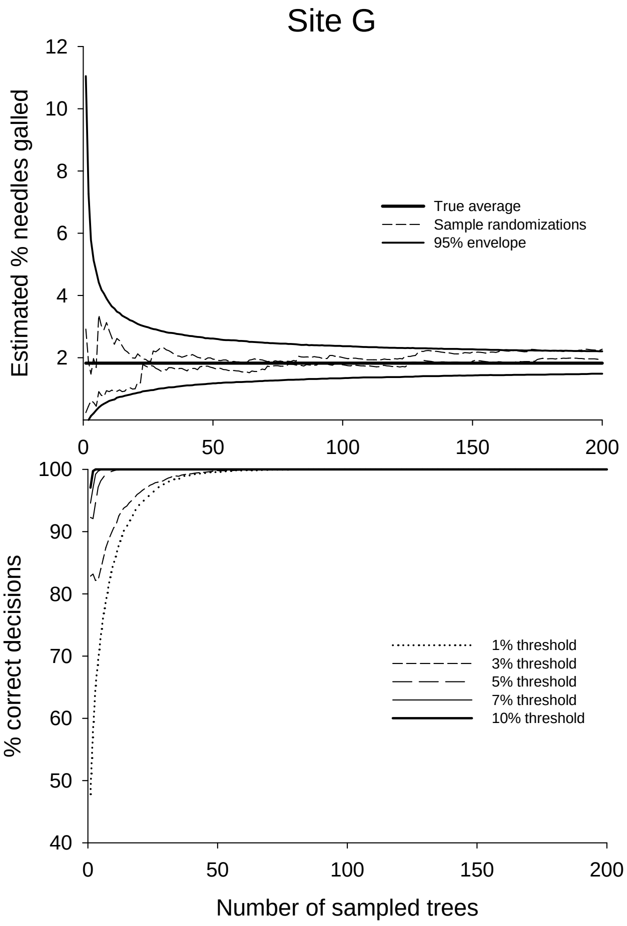

Figure S5. Performance of random sampling (continued).
