# Supplementary material for: A Simulation Approach to Assessing Sampling Strategies for Insect Pests: An Example with the Balsam Gall Midge
Source: PLoS One. 2013 Dec 23;8(12):e82618. doi: 10.1371/journal.pone.0082618 (PMC3871163; doi:10.1371/journal.pone.0082618)
Supplement: Figure S6 — Summary of our analytical approach. Italics refer to R scripts and software available as Software S1, S2, S3, S4. (PDF) [file pone.0082618.s006.pdf]

A. Explore potential for sequential sampling (single-parameterization, SPSS; or local-parameterization, LPSS) using R scripts available in Software S1.

1. Is strong spatial autocorrelation present? → **YES** → Reject SPSS/LPSS → to Chart B*
   (Mantel.R, Variogram.R)

↓

NO

↓

2. Can infestation data be fit to a standard statistical distribution? → **NO** → Reject SPSS/LPSS → to Chart B
   (*Normal.R, NegBin.R)*

↓

YES

↓

choose distribution

↓

3. Do parameter estimates vary among sites? → **NO** → Use SPSS
   (NegBin.R)

↓

YES

↓

Reject SPSS

↓

4. Can local parameters be estimated with reasonable sample sizes? → **NO** → Reject LPSS → to Chart B
   (SubSampleK.R)

↓

YES

↓

Use LPSS

**Or develop more complex methods for sequential sampling with autocorrelation*

Figure S6. Summary of our analytical approach. Italics refer to R scripts and software available as Software S1-S4.

B. Simulate simple non-sequential sampling schemes *(InfestSample1.10)*

1. Does random/ordered/transect sampling converge on true mean? → **NO** → Reject simple sampling → need to develop new methods

↓

YES

↓

2. Does % correct decisions approach acceptable rate for reasonable sample size? → **NO** → 3. Can a modest threshold adjustment correct the problem? → **NO** → Reject simple sampling

↓ (from 3.)

YES → Choose sampling scheme and sample size for desired precision

↓ (from Reject simple sampling)

need to develop new methods

↓ (from 2.)

YES

↓

Choose sampling scheme
and sample size
for desired precision

↓

Apply simple
random/ordered/transect
sampling

Figure S6. Summary of our analytical approach (continued).
