# Supplementary material for: A Simulation Approach to Assessing Sampling Strategies for Insect Pests: An Example with the Balsam Gall Midge
Source: PLoS One. 2013 Dec 23;8(12):e82618. doi: 10.1371/journal.pone.0082618 (PMC3871163; doi:10.1371/journal.pone.0082618)
Supplement: Software S2 — Zipped executable version of software InfestSample version 1.10. (ZIP) [file pone.0082618.s009.zip › InfestSample110/Help/InfestSampleHelp.htm]

# Using InfestSample 1.10

## August 2, 2013

This program is free software: you can redistribute it and/or modify
it under the terms of the GNU General Public License as published by
the Free Software Foundation, either version 3 of the License, or
(at your option) any later version.
This program is distributed in the hope that it will be useful,
but WITHOUT ANY WARRANTY; without even the implied warranty of
MERCHANTABILITY or FITNESS FOR A PARTICULAR PURPOSE. See the
GNU General Public License for more details, http://www.gnu.org/licenses/

Using InfestSample is straightforward. This help document explains each option in the user interface window.

**Input data file:** This is a comma-delimited text file containing the data needed to simulate sampling. Here is an example:

Site,Tree,Percent\_galled,Lat\_m\_46,Long\_m\_67
  
G,1,2.997120516,23118.49659,14914.20635
  
G,2,0.155011603,23134.6152,14914.82073
  
G,3,1.93760167,23148.73969,14913.20296
  
G,4,0.671753859,23166.73905,14908.26089
  
G,5,0.354148594,23179.08395,14905.33023
  
G,6,1.492175529,23194.30776,14900.46457
  
G,7,11.8291901,23210.42638,14899.08066
  
G,8,13.57318727,23212.09482,14883.02336
  
G,9,0.814915452,23199.08744,14884.87346
  
G,10,1.793322222,23185.74659,14881.7097
  
G,11,0.930794498,23171.51873,14881.86484

The first row consists of column headers, and is not actually used by the program, so a dummy string of text is fine. The following rows consist of data, with 5 columns:

1. Site label (here, “G”)
  
2. Sampling unit ID, which should be integer
  
3. Measure of insect attack/infestation for that sampling unit
  
4-5. X and Y coordinates for location of sampling unit (if unavailable, enter dummy data, but transect sampling will not work)

**Output data file:** This is a comma-delimited text file, generated when InfestSample is run, containing estimates of infestation for the simulated sampling. After some header information, it includes a row for each sample size 1, 2, 3.....n where there are n sampling units in the full sample. There are 20 columns:

1. Number of sampling units included
  
2. Estimate of infestation by random sampling
  
3. Another estimate of infestation by random sampling using a different randomization
  
4. The average estimate for all randomizations done, which should equal the true average for large numbers of randomizations
  
5-9: Percent correct decisions for the 5 infestation thresholds
  
10-11. 2.5th and 97.5th percentiles of randomization estimates, which make a 95% confidence interval
  
12. True average infestation (same for all rows, of course)
  
13. Average absolute deviation of randomization estimates from true average
  
14. 95th percentile of absolute deviations
  
15-16. Estimates for sampling ordered by sampling unit ID number, counting up and down respectively
  
17-18. Absolute deviations for ordered sampling, counting up and down respectively
  
19. Estimate for transect sampling
  
20. Absolute deviation for transect sampling.
  
  
If an appropriate file already exists and is to be replaced, select it using the "Browse" button. To create a new file, either type file path and file name directly into the text box, or use the "Change path" button to select a folder (which will appear in the text box as a file path) and then type the file name into the text box after the file path.

**Output map file:** This is another comma-delimited file, generated when InfestSample is run, containing data necessary to generate a map of the included and excluded sampling units for transect sampling. It has four columns:

1. Sampling unit ID
  
2-3. X and Y coordinates of sampling unit
  
4. Inclusion (1) or exclusion (0) of that sampling unit.
  
  
If an appropriate file already exists and is to be replaced, select it using the "Browse" button. To create a new file, either type file path and file name directly into the text box, or use the "Change path" button to select a folder (which will appear in the text box as a file path) and then type the file name into the text box after the file path.

**Infestation thresholds 1-5:** These are values for infestation levels against which estimated infestation is to be judged.

**End sampling units for transect A:** These are the sampling unit IDs that define the start and end points of the belt transects for transect sampling. It works well to specify units at opposite corners of the sampled area.

**End sampling units for transect B:** For sites with obvious sub-site structure, specifying a 2nd transect allows a form of stratified sampling where the transect is run first through one subsite and then through another. If these are left blank, only the main set of transects is used.

**Jitter for transects:** Sampling units are added to the sample along the main transect, then a second transect parallel to the first, then a third parellel to the first (but to the other side). The jitter specifies the distance, in units of the X,Y coordinate system, between adjacent transects.

**Number of randomizations:** Affects only random sampling; randomization is repeated this many times. Unless data sets are very large, 10,000 randomizations takes a trivial amount of execution time but is large enough for excellent performance of the analyses.

  
  
  

**Installing the .NET Framework:** Most versions of Windows should already have installed an element necessary to run InfestSample110: the “.NET Framework”.
However, if necessary, you can acquire this free download at http://www.microsoft.com/en-us/download/details.aspx?id=30653
